# Brazilian guideline for the use of immunobiologicals in chronic rhinosinusitis with nasal polyps ‒ 2024 update

**DOI:** 10.1016/j.bjorl.2024.101394

**Published:** 2024-01-30

**Authors:** Wilma T. Anselmo-Lima, Fabrizio R. Romano, Edwin Tamashiro, Renato Roithmann, Vanessa R.P. Dinarte, Otavio B. Piltcher, Marcel M. Miyake, Marco A. Fornazieri, Marcio Nakanishi, Thiago F.P. Bezerra, Ricardo L.L. Dolci, João F. Mello Jr, Marcus M. Lessa, Richard L. Voegels, Eduardo M. Kosugi, Eulalia Sakano, Fabiana C.P. Valera

**Affiliations:** a Universidade de São Paulo, Faculdade de Medicina de Ribeirão Preto, Departamento de Oftalmologia, Otorrinolaringologia, Cirurgia de Cabeça e Pescoço, Ribeirão Preto, SP, Brazil; b Faculdade de Medicina da Universidade de São Paulo, São Paulo, SP, Brazil; c Universidade de São Paulo (FMRP-USP), Faculdade de Medicina de Ribeirão Preto, Ribeirão Preto, SP, Brazil; d Universidade Luterana do Brasil, Canoas, RS, Brazil; e Faculdade de Medicina de Marília (FAMEMA), Marilia, SP, Brazil; f Faculdade de Medicina da Universidade Federal do Rio Grande do Sul (FAMED-UFRGS), Departamento de Oftalmologia e Otorrinolaringologia, Porto Alegre, RS, Brazil; g Santa Casa de Misericórdia, Hospital de São Paulo, Departamento de Otorrinolaringologia, São Paulo, SP, Brazil; h Universidade Estatual de Londrina, Londrina, PR, Brazil; i Pontifícia Universidade Católica do Paraná (PUCPR), Câmpus Londrina, Londrina, PR, Brazil; j Universidade de Brasília, Faculdade de Medicina, Programa de Pós-Graduação, Brasilia, DF, Brazil; k Universidade Federal de Pernambuco, Recife, PE, Brazil; l Santa Casa de São Paulo, São Paulo, SP, Brazil; m Faculdade de Medicina da Universidade Federal da Bahia, Salvador, BA, Brazil; n Universidade Federal de São Paulo, Escola Paulista de Medicina, Departamento de Otorrinolaringologia e Cirurgia de Cabeça e Pescoço, São Paulo, SP, Brazil; o Universidade Estadual Paulista, Faculdade de Ciências Médicas, Departamento de Oftalmologia/Otorrinolaringologia, Campinas, SP, Brazil

**Keywords:** Biologicals, Monoclonal antibodies, Chronic rhinosinusitis, Nasal polyp

## Abstract

**Introduction:**

Biologics targeting type 2 inflammation have revolutionized the way we treat patients with Chronic Rhinosinusitis with Nasal Polyps (CRSwNP). Particularly in severe and difficult-to-control cases, these drugs have provided a new reality for these patients, allowing for the effective and safe treatment of extensive diseases that were not completely managed with the typical strategy of surgery and topical medications.

**Objectives:**

The experience achieved with the approval of these medications by ANVISA for use in CRSwNP and the knowledge obtained regarding outcomes, adverse effects, and the ideal patient profile prompted the update of the previously published guideline, with a detailed review of the most recent scientific literature, the personal experiences of experts, and the adaptation to the reality of the Brazilian healthcare system, both public and private.

**Results:**

We proposed a new eligibility criterion for biologics in patients with CRSwNP based on four pillars of indication: the impact of the disease on the patient’s life, whether in the presence of specific symptoms or in overall quality of life; the extent of sinonasal disease; the presence of type 2 comorbidities, considering other associated diseases that may also benefit from anti-T2 biologics, and the presence of biomarkers to define type 2 inflammation, especially those associated with worse disease prognoses.

**Conclusions:**

This innovative and pioneering method has two major advantages. First, it ensures a comprehensive evaluation of patients; second, it is flexible, as advancements in our understanding of the disease and changes in cost-effectiveness can be addressed by simply adjusting the required score for indication, without the need to modify the entire evaluation scheme.

## Highlights

• This is the 2024 updated Brazilian Guideline for Biologicals in CRSwNP.
• Our understanding of Biologics has increased.
• A comprehensive score system for the indication of biologics is proposed.
• The higher the score, the stronger the indication for biologics.
• This will help ENT physicians in selecting the appropriated patients.

## Introduction

Biologics targeting type 2 inflammation have changed the way we treat patients with CRSwNP. In severe and difficult-to-control cases, these medications have introduced a new paradigm, allowing for the effective and safe treatment of extensive diseases that were previously not fully managed with the conventional approach of surgery and topical medications. However, due to the high costs associated with these biologics, their indication requires careful and thorough consideration, so as not to overly burden an already overloaded system, while at the same time ensuring that these treatments are indicated for those who genuinely need them.

The experience gained following the approval of these drugs by ANVISA for use in CRSwNP, the insights acquired regarding outcomes and adverse effects, and the ideal patient profile motivated the update of the previously published guideline. This update entailed a comprehensive review of the latest scientific literature, the personal experiences of experts, and alignment with the reality of the Brazilian healthcare system, both public and private.

## Type 2 inflammation in CRSwNP

Formerly known as type 2 helper T-cell inflammation, type 2 inflammation received its name because it is orchestrated by inflammatory mediators produced by type 2 helper T cells (Th2), including cytokines IL-4, IL-5, IL-9, and IL-13, with eosinophils as the main cellular marker, in addition to elevated levels of local or circulating IgE. Further research revealed that other non-Th2 cells, such as type 2 Innate Lymphoid Cells (ILC2), are also responsible for producing these cytokines. This led to the simplification of the term to “type 2” inflammation. ILC2 act as early effectors of type 2 inflammation by releasing IL-5, IL-9, and IL-13 before the allergic response, as their activation is independent of IgE production and its binding to the antigen.^1^

When a mucosal barrier is damaged, a self-limiting immune response is generated, which can be type 1 in cases of viruses, type 2 for parasites, and type 3 for bacteria and fungi. In type 1 response, one of the main cytokines is INF-γ. In type 2, interleukins IL-4, IL-5, and IL-13 play a crucial role, while type 3 involves IL-17 and IL-22.^2^ Each immune response follows a fast pathway through innate lymphoid cells (ILC-1, 2, or 3) and a slow pathway generated by helper T lymphocytes (Th1, TH2, Th17).^2^ In CRS, these responses, whether type 1, 2, or 3, or their combinations, become chronic.^2^

Type 2 response encompasses both innate and adaptive immune responses, aiming to provide protection in mucosal barriers, especially in the defense against parasites and response to allergens.^3^, ^4^, ^5^ Various stimuli can trigger type 2 response, such as the presence of helminths, allergens, bacterial infections, and viruses. This response is characterized by the presence of Th2 lymphocytes, B lymphocytes, IgE-producing plasma cells, eosinophils, basophils, and mast cells, and is associated with various cytokines, including IL-4, IL-5, IL-9, and IL-13. Some cytokines produced and released by the epithelium, such as Thymic Stromal Lymphopoietin (TSLP), IL-25, and IL-33, can initiate or amplify type 2 responses.^3^

**Figure 1:**
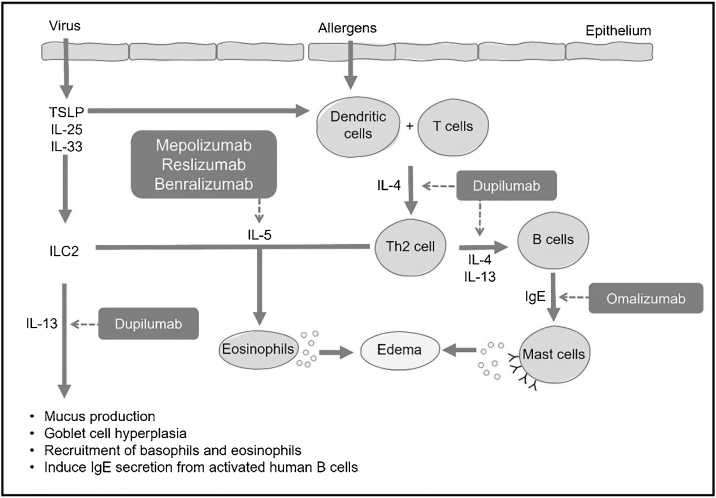
Schematic diagram of type 2 response in the sinonasal mucosa, and the potential local action of therapies involving immunobiologicals (or monoclonal antibodies).

In allergic processes, dendritic cells capture antigens in the mucosa and present them to T lymphocytes, leading to their differentiation into Th2 lymphocytes. These Th2 lymphocytes, in turn, produce and secrete IL-4 and IL-13, prompting naive B lymphocytes to differentiate into specific IgE-producing plasma cells (Fig. 1). Circulating IgE then binds to high-affinity receptors present on mast cells and basophils, and, upon subsequent contact with the same antigen, these cells are activated, releasing histamine, prostaglandins, and various pro-inflammatory cytokines (IL-4, IL-5, and IL-13). These mediators induce mucus production, hyperplasia of goblet cells, and thickening of the basal membrane, as well as create a reverberating cycle, attracting circulating eosinophils to the peripheral organ (Fig. 1).^3^, ^4^

## Effects of type 2 interleukins in CRS

Interleukin-5 (IL-5) is a pro-eosinophilic cytokine that regulates the differentiation and maturation of these cells in the bone marrow. It also induces activation and enhances tissue survival, thereby reducing the degree of apoptosis.^3^, ^6^, ^7^

The effects of IL-4 encompass the differentiation of T lymphocytes into Th2, the induction of B lymphocytes for IgE production, chemotaxis for eosinophils, and recruitment and activation of mast cells and basophils.^3^, ^6^, ^7^ IL-13 is chemotactic for eosinophils, induces B lymphocytes to produce IgE, and activates mast cells and basophils. Additionally, it induces mucus secretion, hyperplasia of goblet cells, and collagen production.^3^, ^6^, ^7^ IL-33 is also a mediator of type 2 inflammation. It binds to surface receptors on Th2 lymphocytes, ILC2, basophils, eosinophils, mast cells, dendritic cells, among others, activating inflammation in the airways. Direct exposure of the airway epithelium to *S. aureus*, for example, increases the expression of IL-33 and TSLP, which induce the production of cytokines such as IL-5 and IL-13, playing an important role in the onset and/or maintenance of type 2 inflammation in CRSwNP.^2^, ^7^

## CRS with type 2 inflammation

In CRS, some phenotypes are characterized by type 2 responses,^2^, ^3^ such as Eosinophilic Chronic Rhinosinusitis with Nasal Polyps (CRSwNPe), Allergic Fungal Rhinosinusitis (AFRS), Atopic Disease of the Central Compartment (ADCC), Eosinophilic Granulomatosis with Polyangiitis (EGPA), and Granulomatosis with Polyangiitis (GPA).

## Eosinophilic chronic rhinosinusitis with nasal polyps: CRSwNPe

This disease primarily affects individuals in the older age group, typically between 30 and 50 years old, and may manifest with acute exacerbations, loss of smell, late-onset asthma, and exhibits a favorable response to oral and topical corticosteroids.

An important subtype, associated with a more reserved prognosis, is AERD, whose diagnostic characteristics include the presence of nasal polyps, asthma, and intolerance to aspirin or Non-Steroidal Anti-Inflammatory Drugs (NSAIDs). The diagnosis of AERD can be established clinically when such findings are evident. However, in some cases, tests such as oral provocation with aspirin or spirometry may be necessary to confirm intolerance to aspirin or NSAIDs and the presence of asthma, respectively.^6^ The primary symptoms include nasal obstruction, rhinorrhea, and severe asthma following the ingestion of NSAIDs. Gastrointestinal symptoms and urticaria may be observed in up to 30% of patients. Generally, the disease manifests after the age of 35, being more frequent in women without a history of atopy.^8^, ^9^, ^10^

Among the phenotypes of CRSwNP associated with type 2 inflammatory response, two of them have a stronger association with IgE-mediated allergy: AFRS and ADCC.

## Allergic fungal rhinosinusitis (AFRS)

RSFA typically affects immunocompetent adolescent or young adult patients who exhibit a strong atopic component. Although there is still no consensus on its diagnosis, the most accepted criteria are the 5 elements established by Bent and Kuhn^11^: (1) Presence of chronic rhinosinusitis with nasal polyps; (2) IgE-mediated hypersensitivity to fungi; (3) Eosinophilic mucin (often with intense eosinophilic degranulation and the formation of Charcot-Leyden crystals); (4) Presence of non-invasive fungal structures, and (5) Radiological findings characteristic of fungal presence, such as compact hyperdensities in the paranasal sinuses (combination of various metals concentrated by fungi).^2^ The disease can be treated with multiple courses of oral and topical corticosteroids, antibiotics, and surgery.

## Atopic disease of the central compartment (ADCC)

Although atopy is not universally associated with CRS, in some forms, there is a clear association between the two. In these cases, recently described as ADCC,^8^ CRS typically manifests as of the age of 18 alongside other allergic manifestations, such as allergic rhinitis, dermatitis, and asthma, which are usually present since childhood. These patients generally do not exhibit olfactory alterations. Upon endoscopic examination, the presence of polypoid changes in the middle and upper turbinate’s and the posterior superior septum is noteworthy, while the mucosa of the paranasal sinuses appears normal or with few alterations on computed tomography.^8^ In ADCC, there is usually a good response to treatments involving topical and oral corticosteroids.

Two forms of CRS secondary to autoimmune processes have been increasingly explored regarding the use of biologics: Eosinophilic Granulomatosis with Polyangiitis (EGPA) and Granulomatosis with Polyangiitis (GPA), both of which still have undetermined etiologies.

## Eosinophilic granulomatosis with polyangiitis (EGPA)

EGPA, otherwise known as Churg-Strauss syndrome, is a small vessel vasculitis that differs from other vasculitides by the presence of severe asthma and extremely elevated eosinophils (systemic and local) in adults. The sinonasal manifestation usually occurs in the early stages of the disease (prodromic or eosinophilic), and is clinically manifested as allergic rhinitis, recurrent rhinosinusitis, or CRSwNP. Laboratory findings are nonspecific and include eosinophilia above 10% or more than 1000 cells/μL, elevated inflammatory activity markers (erythrocyte sedimentation rate and C-reactive protein), and increased serum IgE, Rheumatoid Factor (RF), and Antinuclear factor (ANF). It is associated with renal and neuropathic changes, with biopsy revealing vasculitis. Although it is an autoimmune disease, only 30%–40% of patients have a positive p-ANCA.^2^, ^3^

According to the update of the American College of Rheumatology^12^ for the classification of EGPA, two criteria have been established: (1) Clinical: Obstructive pulmonary disease +3, presence of nasal polyps +3, and mononeuritis multiplex +1; (2) Laboratory and histopathological: eosinophilia ≥ 1 × 10^9^/L: +5, biopsy demonstrating extravascular inflammation with predominance of eosinophils: +2, C-ANCA or anti-PR3: −3, hematuria: −1. The diagnosis of EGPA is confirmed when the final score is ≥6.

## Granulomatosis with polyangiitis (GPA)

Granulomatosis with Polyangiitis (GPA), formerly known as Wegener’s granulomatosis, is defined as a chronic autoimmune inflammatory disease of unknown etiology characterized by necrotizing granulomatous lesions and systemic vasculitis of small and medium-sized vessels and is strongly associated with Anti-Neutrophil Cytoplasmic Antibodies (ANCA).^2^

Several *in vitro* and *in vivo* studies have indicated that ANCA induces systemic vasculitis by binding to and activating neutrophils, leading to the release of oxygen radicals, lytic enzymes, and inflammatory cytokines. ANCA can also induce the formation of immune complexes and directly adhere to endothelial cells, causing vasculitis.^13^ Although c-ANCA (anti-proteinase-3) is highly specific for GPA, the initial trigger may be an infection or other environmental factors, possibly combined with genetic susceptibility.^2^, ^14^

GPA is defined when 2 out of the 4 diagnostic criteria established by the American College of Rheumatology are met^14^: (1) Abnormalities in routine urine examination (presence of hematuria or more than 4 red blood cells per high-power field); (2) Alterations in chest X-Rays (nodules, cavities, inflammatory infiltrates); (3) Oral ulcers or nasal discharge, and (4) Biopsy indicating granulomatous inflammation. Currently, some monoclonal antibodies are being used in the treatment of GPA, which will be discussed further.

## Biologicals

Several biological products are being studied for use in respiratory diseases, such as anti-IgE (omalizumab), anti-IL-5 (mepolizumab, reslizumab, benralizumab), anti-IL-4, and anti-IL-13 (dupilumab), among others.

## Omalizumab (Anti-IgE)

Omalizumab is a monoclonal anti-IgE antibody that was approved by the FDA in 2003 for the treatment of moderate-persistent uncontrolled allergic asthma with inhaled corticosteroids, being the first biologic used for type 2 inflammatory diseases.^13^, ^15^, ^16^, ^17^ Studies have shown improvements in asthma control, a reduction in the number of exacerbations, and a decreased need for oral corticosteroids and rescue medications.^15^ In January 2021, the Brazilian National Health Surveillance Agency (ANVISA) approved omalizumab for use in Chronic Rhinosinusitis with Nasal Polyps (CRSwNP) as a complementary treatment to intranasal corticosteroids in adult patients above 18 years old.

## Mechanism of action

Omalizumab is a monoclonal anti-IgE antibody that recognizes and binds to the high-affinity Fc receptor of IgE in a variety of inflammatory cells in the blood and interstitial fluid, blocking the IgE-mediated inflammatory cascade and significantly reducing serum concentrations of free IgE in a dose-dependent manner.^16^, ^18^, ^19^ Omalizumab also has a secondary effect, resulting in the downregulation of the Fc receptor on basophils, mast cells, and dendritic cells, leading to a reduction in the release of inflammatory mediators, in addition to reducing serum free IgE.^18^, ^20^

By selectively binding to circulating IgE, omalizumab prevents the binding of IgE to mast cells and other effector cells. Without IgE bound to the surface, these cells are unable to recognize allergens, thus preventing cellular activation by antigens and, consequently, subsequent allergic/asthmatic symptoms. The reduction in the density of the IgE receptor on effector cells results in a significant improvement in airway inflammation parameters.^17^

After subcutaneous administration, omalizumab is slowly absorbed, reaching peak serum concentrations after 7–8 days, on average, with a terminal half-life of 26 days.^21^, ^22^

## Review of the use in other respiratory diseases

In adults and adolescents (≥12 years old) with moderate-to-severe allergic asthma, the subcutaneous administration of omalizumab as adjuvant therapy with inhaled corticosteroids improved the number of asthma exacerbations, the use of rescue medication, the asthma symptom score, and Quality of Life (QOL) compared to the placebo group during 28- and 32-week double-blind trials. Additionally, the concomitant use of inhaled corticosteroids significantly decreased in patients receiving omalizumab. Overall, the results obtained in the extension studies demonstrated that the beneficial effects of omalizumab were maintained over a total period of 52-weeks.^17^

One approach described in the literature for patients with Aspirin-Exacerbated Respiratory Disease (AERD) is the use of omalizumab, in addition to the patient’s usual medications. The mechanism behind this involves the suppression of PGD2 production and the overproduction of CysLT, rather than the attenuation of eosinophilic inflammation. Omalizumab suppresses mast cell activation in patients with refractory urticaria and is also considered effective as a mast cell stabilizer for AERD patients.^10^, ^23^ Two case reports showed clinical improvements and loss of aspirin sensitivity in patients with AERD and severe asthma after treatment with omalizumab.^24^, ^25^

## Review of the use in CRS (efficacy)

Given the high concentrations of IgE in the mucosa of nasal polyp tissue and its relevance to the severity of the disease and comorbidities, strategies to antagonize IgE may be relevant in patients with CRSwNP.^26^

In a randomized, double-blind, placebo-controlled Phase II trial conducted by Gevaert et al. (2013)^27^ in patients with CRSwNP and associated asthma, patients were selected to receive 4–8 subcutaneous doses of omalizumab (n = 16) or placebo (n = 8) for 16-weeks.^27^ A significant reduction was observed in the nasal polyp score (a measurement scale that assesses the size, location, and degree of obstruction of the polyp) and the Lund-MacKay tomographic score (quantitative measurement of inflammation by computed tomography of the paranasal sinuses) of the omalizumab group compared to the placebo group. In addition, omalizumab had a significantly greater beneficial effect on upper airway symptoms, including nasal congestion, anterior rhinorrhea, and loss of smell, as well as lower airway symptoms, such as wheezing and dyspnea. It is important to note that omalizumab was also associated with improved quality of life scores in patients with CRSwNP and asthma.^16^, ^18^, ^27^ In another randomized, double-blind, placebo-controlled clinical trial in refractory CRSwNP, by Pinto et al. (2010), the patients were randomized to receive either omalizumab or placebo for 6-months.^28^ Omalizumab treatment was associated with a significant improvement in quality of life (SNOT-20) at various time intervals, including 3, 5, and 6 months of follow-up compared to baseline; in contrast, no significant changes were observed in the control group.^16^, ^28^

Rivero and Liang (2017),^29^ in a systematic review evaluating studies on anti-IgE therapy, did not report a statistically significant reduction in the nasal polyp score compared to the placebo group, although there was a tendency of improvement.^29^ The post-hoc analysis of studies where patients had concomitant severe asthma as a formal inclusion criterion showed a statistically significant reduction in the nasal polyp score. The authors concluded that anti-IgE therapy reduces the nasal polyp score in patients with associated severe asthma.^29^

In the systematic review carried out by Tsetsos et al. (2018),^30^ the authors compared the results of the randomized clinical trials conducted by Gevaert et al. (2013)^27^ and by Pinto et al. (2010),^28^ which investigated the efficacy of omalizumab as an alternative therapeutic option in patients with CRSwNP and associated asthma.^30^ Clinical improvement was measured in both trials through the total nasal polyp score, pre- versus post-treatment sinus opacification on computed tomography of the paranasal sinuses, quality of life measures, and peak nasal inspiratory flow, nasal inspiratory flow, and olfaction (UPSIT).^18^, ^30^ The clinical trial conducted by Pinto et al. (2010)^28^ did not show statistically significant changes in any of the mentioned categories. The trial carried out by Gevaert et al. (2013),^27^ on the other hand, exhibited significant improvements in all measurements except for nasal inspiratory flow and olfaction.^18^, ^30^ It is noteworthy that the study by Gevaert et al. (2013)^27^ had some potential limitations, including a limited number of participants (n = 24) and higher baseline eosinophilic inflammation in individuals treated with placebo, despite the randomization.^18^, ^30^ The placebo group of the study also had a 50% dropout rate. The trial conducted by Pinto et al. (2010)^28^ also presented limitations regarding the number of enrolled participants, emphasizing the need for a trial with a larger number of individuals.^18^, ^30^ Two other systematic reviews also pointed out the need for additional evaluation of the efficacy of anti-IgE therapy in these patients.^31^, ^32^ In addition to establishing clinical benefit, other obstacles need to be overcome for omalizumab to be officially incorporated into the effective therapy of CRSwNP. Cost-effectiveness was not evaluated in any of the mentioned studies, despite being a crucial factor in managing this disease.^30^ Gevaert et al. (2020) published the results of the Phase III POLYP 1 and 2 trials.^33^ The authors demonstrated that patients treated with omalizumab achieved statistically significant improvements in the mean nasal polyp score (POLYP 1: −1.08 vs. 0.06; *p* < 0.0001, POLYP 2: −0.90 vs. −0.31; *p* =  0.014) and in the daily nasal congestion score (NCS – POLYP 1: −0.89 vs. −0.35; *p* =  0.0004, POLYP 2: −0.70 vs. −0.20; *p* =  0.0017) compared to the placebo group on week 24. All patients received intranasal corticosteroids (mometasone) as baseline therapy. In both trials, patients treated with omalizumab showed significant improvements in the Nasal Polyp Score (NPS) and in the Nasal Congestion Score (NCS) since the first assessment on the fourth week when compared to the placebo. Improvements were observed in the assessment of health-related quality of life (SinoNasal Outcome Test-22 ‒ SNOT-22), the University of Pennsylvania Smell Identification Test (UPSIT), the Total Nasal Symptom Score (TNSS), and sense of smell. Additionally, decreased posterior and anterior rhinorrhea were also observed. According to the authors, omalizumab was generally well-tolerated, and its safety profile was consistent with previous studies.^33^

In 2022, Gevaert et al. conducted an open study to evaluate the continuity of efficacy, safety, and durability of the response to omalizumab in adults with CRSwNP who completed the POLYP 1 or 2 trials.^34^ After 24 weeks of omalizumab or placebo in POLYP 1 and 2, the patients (n = 249) received open-regimen omalizumab in conjunction with nasal corticosteroid spray (mometasone) for 28-weeks and were subsequently followed up for 24-weeks after omalizumab discontinuation. Patients who continued with omalizumab experienced additional improvements in primary and secondary outcomes over 52-weeks. Those who switched from placebo to omalizumab showed favorable responses in primary outcomes up to week-52, which were similar to POLYP 1 and 2 on week-24. After discontinuation of omalizumab, the patients’ scores gradually worsened over the 24-week follow-up period but remained better than pretreatment levels for both groups. The authors concluded that the efficacy and safety profile observed in this study endorsed the prolonged treatment with omalizumab for up to 1-year for patients with CRSwNP with an inadequate response to nasal corticosteroids.^34^

Damask et al. (2022) assessed the efficacy of omalizumab therapy versus placebo in patients with CRSwNP from the replicated POLYP 1 and POLYP 2 trials, who were grouped by inherent patient characteristics to determine therapy response.^35^ Pre-specified subgroups from POLYP 1 and POLYP 2 were examined, and included blood eosinophil count at the beginning of the study (≤300 or >300 cells/μL), prior sinus surgery (yes or no), asthma status (yes or no), and aspirin sensitivity status (yes or no). The subgroups were analyzed regarding the subgroup-specific adjusted mean difference (95% Confidence Interval/Omalizumab-placebo) in the change from baseline on week-24 in the Nasal Congestion Score (NCS), the Nasal Polyp Score (NPS), the SNOT-22 results, the Total Nasal Symptom Score (TNSS), and the University of Pennsylvania Smell Identification Test (UPSIT). This subgroup-specific adjusted mean difference in the change from baseline in NCS, NPS, SNOT-22, TNSS, and UPSIT on week-24 consistently favored omalizumab treatment over placebo in patients with blood eosinophil counts ≤300 and >300 cells/μL, with or without prior sinus surgery, asthma, and aspirin sensitivity. The authors concluded that the data obtained in the study suggest broad efficacy of omalizumab treatment in patients with CRSwNP regardless of underlying patient factors, including those with elevated levels of eosinophils and who underwent previous surgery, which are more associated with high recurrence. In light of these results, the authors suggest that the improvements across all population subgroups may be explained by a shared underlying pathophysiological process of IgE-mediated inflammation.^35^

A retrospective non-randomized real-life interventional study was conducted by Maza-Solano et al. (2023), from July 2016 to November 2020, evaluating patients with CRSwNP and allergic asthma.^36^ The study participants were divided into four subgroups based on surgical intervention and/or treatment administered. In Group 1 (Omalizumab + endoscopic sinus surgery), the patients had a history of previous endoscopic sinus surgery and were also treated with omalizumab; in Group 2 (Omalizumab), the patients were treated with omalizumab without a previous history of endoscopic sinus surgery; in Group 3 (endoscopic sinus surgery), the patients underwent endoscopic sinus surgery but were not treated with omalizumab; and in Group 4 (control), the patients did not undergo surgery, nor did they receive omalizumab. The authors demonstrated that complementary therapy with omalizumab in patients with prior endoscopic sinus surgery provided greater improvement than the two therapies separately, both in quality of life (SNOT-22) and in the endoscopic results (nasal polyp score and modified bilateral Lund-Kennedy score), as early as the 16th week of treatment; this improvement continued to be observed over a 2-year follow-up period. The effects of both approaches may complement each other and lead to better control of the underlying inflammatory disease, with a decrease in mucous secretion, edema, and long-term polyp recurrence.^36^ The often-proposed differentiation of patients with nasal polyps into non-allergic individuals with elevated blood eosinophils for choosing anti-IL-5 therapy, and allergic patients for anti-IgE treatment, is not supported by evidence. Omalizumab worked at least as well in non-allergic individuals compared to allergic individuals. While omalizumab is known to decrease free IgE antibodies, it is still unclear which biomarker is crucial for the clinical effect in nasal polyps. No currently used biomarker, such as blood eosinophils or total serum IgE, has shown to assist in the selection or prediction of responses to immunobiologicals.^37^ In the study by Gevaert et al. (2013) on patients with CRSwNP and associated asthma, omalizumab significantly decreased total nasal polyp scores and sinus opacification and improved nasal symptoms, including olfaction, in both allergic and non-allergic individuals.^27^ Additionally, omalizumab significantly improved asthma symptoms and quality of life, irrespective of the presence of allergy.^38^ Recent observations demonstrating efficacy in patients with non-allergic asthma support these findings.^38^

The effect of anti-IgE biologic therapy has also been explored in subsets of patients with Eosinophilic Granulomatosis with Polyangiitis (EGPA).^18^ Observational reports have shown that patients with EGPA benefit from omalizumab therapy in the context of asthma and associated sinus disease. The mechanism of omalizumab does not target the underlying vasculitis, thus contributing to the pathophysiology of EGPA. However, it is believed that the secondary effect of omalizumab, which leads to the indirect apoptosis of eosinophils, helps improve clinical symptoms in patients with specific clinical manifestations. A retrospective review of 17 patients with refractory or relapsing asthma and EGPA treated with omalizumab evidenced a reduction in prednisone dosage to less than 7.5 mg/day but did not demonstrate a decrease in average eosinophil levels.^39^

Omalizumab has been shown to be clinically beneficial in patients with moderate-to-severe asthma and associated AFRS.^40^ Mostafa et al. (2020) compared a single postoperative injection of omalizumab with twice-daily intranasal corticosteroid spray for 6-months in patients diagnosed with AFRS, and the patients were evaluated at 4-week intervals for 6-months. Twenty patients were included in the study and randomly divided into two groups: 10 patients who received a single subcutaneous injection of omalizumab (150 mg) 2-weeks after surgery; 10 patients who received nasal spray containing budesonide or mometasone furoate (100 μg) twice daily for 6-months, starting 2-weeks postoperatively. Both treatments were effective at the end of the 24-week follow-up, but the omalizumab group showed a more significant endoscopic and clinical response, especially in allergic symptoms such as sneezing, itching, and nasal discharge. No significant side effects were observed in either group^21^ (Table 1).

**Table 1:** Clinical trials on the efficacy of Omalizumab in CRSwNP and AFRS.

Study	Method	Participants	Intervention	Outcomes/Results
Mostafa (2020) ^21^	BRCT	20 patients with AFRS	Omalizumab single dose of 150 mg two weeks post-surgery (n = 10)	Significant improvement in both groups, but the group taking Omalizumab showed a better clinical and endoscopic response.
			Topical corticosteroid 2× daily for 6-months starting 2-weeks after surgery (n = 10)	
Pinto et al. (2010) ^28^	DBPCCT	14 patients with CRSwNP/CRSsNP	Omalizumab at the beginning of the study and every 4-weeks for 24-weeks (n = 7)	No statistically significant alterations were observed in any of the outcomes, except for an improvement in SNOT-20 in several time intervals in the Omalizumab group.
			Placebo for 24-weeks (n = 7)	
Gevaert et al. (2013) ^27^	DBPCCT	24 patients with CRSwNP and asthma	Omalizumab maximum dose of 375 mg subcutaneously (4–8 subcutaneous doses for 16-weeks (n = 16)	Significant improvements were observed in all outcomes in the Omalizumab group, except for nasal airflow and olfaction.
			Placebo for 16 weeks (n = 8)	
Gevaert et al. (2020) ^33^	DBPCCT ‒ Phase 3	Polyp 1 138 patients with CRSwNP	Omalizumab 75–600 mg per subcutaneous injection every 2 or 4 weeks, depending on the total IgE serum levels and body weight for 24 weeks (n = 134)	Omalizumab was well tolerated and significantly improved the endoscopic and clinical results of the patients with CRSwNP with an adequate response to intranasal corticosteroids. When compared to the Placebo group.
		Polyp 2 127 patients with CRSwNP	Placebo for 24 weeks (n = 138)	

CRSwNP, Chronic Rhinosinusitis with Nasal Polyps; CRSsNP, Chronic Rhinosinusitis without Nasal Polyps; BRCT, Blind Randomized Clinical Trial; AFRS, Allergic Fungal Rhinosinusitis; DBPCCT, Double-Blind Randomized Placebo-Controlled Clinical Trial; SNOT-20, SinoNasal Outcome Test-20.

## Indications

Omalizumab is used for the treatment of moderate-to-severe persistent allergic asthma in adults and children (above 6-years of age) whose symptoms are inadequately controlled with inhaled corticosteroids. Safety and efficacy have not been established for other allergic conditions. Omalizumab is also indicated as additional therapy for adult and pediatric patients (above 12-years of age) with chronic spontaneous urticaria refractory to treatment with H1 antihistamines.^19^ In 2021, the Brazilian National Health Surveillance Agency (ANVISA) approved omalizumab for use in Chronic Rhinosinusitis with Nasal Polyps (CRSwNP) in adults (18-years old or above) who are already receiving intranasal corticosteroids but whose symptoms are not well controlled by these medications.^19^

## Posology

Omalizumab is slowly absorbed after subcutaneous administration, and the average elimination half-life is 26-days, allowing for infrequent drug administration.^4^ Omalizumab is administered by subcutaneous injection every 2–4 weeks, with the dosage based on the IgE baseline serum levels (IU/mL), measured before the start of treatment, and body weight (kg).^18^

Since omalizumab does not bind to IgE that is already bound to receptors on effector cells, the onset of clinical activity is somewhat delayed. Clinical trials have shown benefits compared to placebo after 4-weeks of therapy, although maximum effects may take longer.^17^

Systemic or inhaled corticosteroid therapy should not be abruptly discontinued upon the initiation of omalizumab therapy. Instead, a gradual reduction of corticosteroid dosage should be attempted over several weeks under medical guidance.^17^

## Safety

Omalizumab has generally been well-tolerated in adolescents and adults with allergic asthma in clinical trials.^17^ It should be administered in a clinical setting due to a 0.2% risk of anaphylaxis.^18^ Previously, there were concerns that Omalizumab might be associated with malignancy; however, data from the prospective cohort study EXCELS suggest that this is not the case.^41^ In 2014, the FDA added cardiovascular risks to the omalizumab label. The most common reactions were upper respiratory tract infection, injection site reactions, and headache, although the incidence of adverse events with Omalizumab was similar to that with placebo.^17^, ^18^ The most common local reactions with subcutaneous omalizumab include bruising, redness, warmth, and itching. Most injection site reactions occur within 1-h after medication administration, and their frequency generally decreases with the continued use of the drug.^17^ Although causal relationships have not been demonstrated, diseases similar to eosinophilic vasculitis, such as EGPA, have also been reported with omalizumab, usually with concomitant reduction of corticosteroid therapy.^29^ Finally, opportunistic infection with herpes zoster and helminthic infections are theoretical risks; therefore, monitoring these infections should be conducted at the physician’s discretion.^29^

## Mepolizumab, reslizumab, and benralizumab (Anti-IL-5)

### Mechanism of action

IL-5 is a cytokine that plays a crucial role in the activation, differentiation, chemotaxis, and survival of eosinophils.^42^, ^43^ As IL-4 and IL-13, IL-5 is a typical marker of type 2 inflammatory response and is increased in a significant proportion of patients with CRSwNP. However, in some populations, such as the Chinese, its levels are not as elevated as in Caucasian populations.^44^

Mepolizumab and Reslizumab are both monoclonal antibodies antagonists of IL-5. They bind to IL-5 inhibiting its signaling and, consequently, reducing the production, maturation, and survival of eosinophils.^45^, ^46^ Mepolizumab inhibits the bioactivity of IL-5 with nanomolar potency by blocking the binding of IL-5 to the alpha chain of the cytokine’s receptor complex expressed on the cell surface of eosinophils, thereby inhibiting IL-5 signaling and reducing eosinophil production and survival.^47^ Reslizumab specifically binds to IL-5 and interferes with the binding of IL-5 to its cell surface receptor.^48^

Benralizumab is a monoclonal antibody that targets the IL-5 receptor. It binds to the alpha subunit of the human Interleukin-5 receptor (IL-5Rα) with high affinity (16 pM) and specificity. The IL-5 receptor is expressed specifically on the surface of eosinophils and basophils. The absence of fucose in the Fc domain of benralizumab results in high affinity (45.5 nM) to Fc RIII receptors on effector immune cells such as natural killer cells, leading to the apoptosis of eosinophils and basophils through increased antibody-dependent cellular cytotoxicity.^49^

## ANTI-IL-5 in respiratory diseases

### In asthma

Currently, there is a predominance of studies related to the use of anti-IL-5 in asthma when compared to its use in CRS.^50^, ^51^ In this context, Cochrane’s systematic review^52^ reveals the robustness of the available evidence, despite some variability in the severity of cases, outcomes, and follow-up time. All of the anti-IL-5 biologics analyzed showed responses considered significant for different outcomes. For example, they reduced asthma exacerbations in approximately 50% of patients using standard treatments (inhaled corticosteroids) without adequate prior disease control. Few studies have assessed the impact of these medications on the quality of life of patients with eosinophilic asthma. In these studies, the impact was modest, and when studied specifically using instruments related to quality of life and asthma, they did not reach significant values. From a laboratory perspective, the treatments led to a reduction or even zeroing of serum eosinophils, nevertheless these findings did not always translate into the same amount of clinical impact. Lung function tests also showed results with a significantly favorable difference for the biologics, despite being also of lesser magnitude.

In absolute terms, treated patients did not achieve improvement percentages greater than 60%.^50^, ^51^, ^52^ At this moment, it remains unclear how many patients need to be treated for such effects to be achieved, as well as the long-term impact of this type of therapy. However, recent studies, analyzing real-life results in more specific populations within the universe of asthmatic patients, have already shown more expressive results. Supporting this observation, there is an insightful review on the trajectory of Mepolizumab, from its initial studies to more recent publications, where it becomes evident that the success of this medication is not solely attributed to the treated population having a type 2 inflammatory disorder, but rather to a constant improvement in the phenotypic differentiation of patients. This improvement, along with the evaluation of more specific outcomes, contributes to achieving more significant results.^53^

Recent systematic reviews have indicated that various anti-IL-5 therapies have demonstrated results in reducing exacerbations, minimizing the use of oral corticosteroids, and increasing Forced Expiratory Volume (FEV1) in practice (real-life studies). These findings for patients with severe eosinophilic asthma closely align with outcomes observed in individual clinical trials for different types of medications within this line.^54^

In summary, the most recent publications and, specifically, real-life systematic reviews, show that anti-IL-5 therapies not only should but must be considered for patients with difficult-to-manage severe eosinophilic asthma.^55^

## In chronic rhinosinusitis

As outlined in the first published Brazilian guideline, the initial evidence concerning the potential impact of this type of medication on chronic rhinosinusitis is derived from in *vitro* studies. These studies involved the injection of anti-IL-5 into nasal polyps of Caucasian patients, revealing an increase in eosinophil apoptosis and a concurrent decrease in their tissue concentration.^45^

In 2006,^56^ Gevaert et al. were the first to investigate anti-IL-5 therapy for CRSwNP. In their study, the improvement in the nasal polyp score was approximately 50% in the arm treated with Reslizumab. Other authors correlated better results with higher pretreatment levels of nasal IL-5. That same study revealed a rebound eosinophilia after the end of treatment at variable times, depending on the administered dose. This phenomenon was not identified after discontinuation of Mepolizumab treatment.^44^

Meanwhile, in 2017, when the first ABR guideline on biologics was published, several Randomized Clinical Trials (RCT) specifically evaluating anti-IL-5 drugs for CRS were also published. All of them demonstrated that different anti-IL-5 biologics promoted improvement compared to the placebo arm in various evaluated parameters, including quality of life, nasal obstruction, the need for systemic corticosteroid use to relieve nasal symptoms, olfaction, polyp size, opacification on computed tomography and the need for polyp surgery.^57^, ^58^, ^59^ In fact, only Benralizumab and Mepolizumab were analyzed for this purpose. In addition, several studies primarily evaluating asthma showed that patients obtained significant improvements in upper airway outcomes with all three options blocking IL-5.

Although Mepolizumab therapy also shows overall statistically significant benefits in relation to polyp size scores and tomographic disease extension, the percentage of improvement did not exceed 60% of the treated patients.^44^

Bachert et al., in 2017, while investigating anti-IL-5 therapy based on pre-established clinical criteria, identified a significant reduction in the need for new surgical treatments in the Mepolizumab-treated arm compared to the placebo. In absolute numbers, the Mepolizumab treatment arm showed a 30% reduction in the need for surgery compared to 10% of patients receiving placebo over a 9-week evaluation period.^60^ An essential aspect that this study could not answer, possibly due to its follow-up time, was regarding the duration of the benefits conferred by this form of treatment.

Previously described RCTs and systematic reviews showed that blocking the inflammatory response related to the effects of IL-5 clearly had an effect on decreasing systemic and nasal eosinophilia.^60^, ^61^ However, larger studies involving eosinophilic CRS are still needed, especially to define which subgroups of patients will show better responses, in order to minimize resource waste and maximize the effects of anti-IL-5 therapy, thus defining the real role of these drugs in CRS (Table 2).

**Table 2:** Clinical trials on the efficacy of anti-IL-5 in CRSwNP.

Study	Method	Participants	Intervention	Outcomes/Results
Gevaert et al. (2006) ^56^	DBPCCT	24 CRSwNP Reslizumab arm: dose 3 mg/kg Placebo arm: dose 1 mg/kg	Reslizumab single EV dose (30 min) or placebo	No difference in safety and tolerance between arms
	Two centers		Controls after 48 h and 1, 2, 4, 8, 12, 16, 20, 24, 28, 32, 36 weeks	Significant reduction in blood eosinophiles in the medication arm with return to baseline on week 12. Rebound eosinophilia on week 24 (1 mg/kg) and week 32 (3 mg/kg)
				Reduction in polyp volume Only in patients with increased levels of nasal IL-5
Gevaert et al. (2011) ^44^	DBPCCT	30 CRSwNP	2 injections with a 28-day interval	Significant improvement in the nasal polyp score and in the tomographic score in 12/20 (mepolizumab arm) compared to the placebo arm with 1/10
		20 Mepolizumab 750 mg		
		10 placebo		
Bachert et al. (2017) ^60^	DBPCCT	105 CRSwNP with recurrent disease	Mepolizumab (750 mg of 30‒30 days), 6 doses	Significant difference in the need for surgical intervention on week 25
		54 Mepolizumab	Placebo 30‒30 days, 6 doses	Mepolizumab arm: 16 patients (30%)
		52 placebo		Placebo arm: 5 (10%)
				Significant reduction in polyp VAS; endoscopic score, symptoms, SNOT-22, on behalf of the arm
				Mepolizumab
				Absence of difference in olfaction between arms
Bachert et al. (2022) ^58^	DBPCCT	413 CRSwNP	30 mg SC 4‒4 weeks 3 initial doses followed by 30 mg SC 8‒8 weeks, both groups of which had intranasal corticosteroids	Benralizumab reduced the nasal polyp score 0.570 (*p* < 0.001)
		207 Benralizumab vs. 206 placebo		Benralizumab reduced the nasal obstruction score by 0.270 (*p* < 0.005)
				No difference in time of first indication for surgery or SNOT-22
Han JK et al. (2021) ^59^	DBPCCT	407 CRSwNP (all with at least one CENS)	100 mg Mepolizumab SC 4‒4-weeks, 52-weeks vs. placebo, SC, 4‒4 weeks, 52-weeks	Reduction in nasal polyp score (*p* < 0.0001)
		206 Mepo. (189 completed the study) vs. 201 placebo (184 completed)	69 Mepo. vs. 65 placebo were still followed up for 76 weeks	Visual nasal obstruction scale (*p* < 0.0001)
				Occurrence of new surgeries and significantly lower systemic corticosteroid use in the Mepo. arm, as well as olfaction, nasal inspiratory flow, SNOT-22

Bachert et al. (2022), in a recent study comparing 207 patients in the medication arm against 206 receiving placebo, showed that Benralizumab, which currently lacks an established indication for CRS, exhibited a significant reduction in the Polyp Score (NPS) when comparing baseline values and at 40-weeks. However, in the SNOT-22 (SinoNasal Outcome Test 22), the times elapsed until the first surgical intervention and/or the use of systemic corticosteroids were not statistically different between the two arms. Olfactory disturbance was significantly reduced among treated patients and subgroup analyses indicated the influence of the presence of asthma, the number of previous polyp surgeries, body mass index, and the initial number of serum eosinophils on treatment effects. Similar to other anti-IL-5 drugs, no significant adverse events occurred during the follow-up period.^58^

Another RCT also evaluated Benralizumab in CRS, with a smaller number of patients and a shorter follow-up period (20-weeks). The percentage of patients with improvements in outcomes was similar. Nevertheless, this study is noteworthy for the high percentage of patients with improved olfaction in the treatment arm (80%) and the lack of statistical significance compared to placebo in the reduction of polyp size.^62^

Mepolizumab, the only anti-IL-5 with an established indication and approval for CRS treatment, was assessed through the SYNAPSE study, where among 854 patients, 407 in the Intention-To-Treat (ITT) population were randomized, with 206 receiving Mepolizumab and 201 in the placebo arm, for a 52-week follow-up period. Among the characteristics of the evaluated population, the difference compared to other studies was that all patients had undergone at least one previous surgical intervention, which, according to the authors, determined a sample with more severe sinonasal disease. The total polyp score and visual analog scale of nasal obstruction were evaluated as primary outcomes. With significant differences compared to placebo, such as a 60% reduction of more than 3 points in the polyp score compared to 36% in the placebo arm, a 30-point reduction in SNOT-22 compared to 14 and the absence of statistical difference in adverse events between the two arms, the authors concluded that Mepolizumab is effective in treating CRSwNP and should be considered an option for managing these patients.^59^

Regarding the need for Systemic Corticosteroid (SC) use in CRSwNP, Chupp et al. (2023), while evaluating Mepolizumab vs. placebo treatment, observed better treatment responses irrespective of previous SC use. By week 52, the likelihood of using SC for nasal polyps was lower with Mepolizumab than in the placebo arm, regardless of prior sinus surgeries, blood eosinophil count, or comorbidities. Thus, in severe CRSwNP, Mepolizumab exhibits a systemic effect that diminishes corticosteroid use, being associated with clinical benefits regardless of previous SC use.^63^

Recent studies have evaluated real-life results, assessing the impact of anti-IL-5 on CRS treatment. Silver J et al. found that the use of systemic corticosteroids was lower in all evaluated cohorts after the use of Mepolizumab in severe asthma and CRS in the United States. The real-life clinical use of Mepolizumab showed benefits to patients with comorbidities, with a greater impact on those with severe asthma plus CRS (comorbidity) plus sinonasal surgery.^64^

When evaluating the treatment of patients with severe CRSwNP with Mepolizumab, real-life studies have demonstrated a significant reduction in symptoms, polyp scores, blood eosinophils, and systemic corticosteroid use, improving the quality of life in these patients regardless of the presence or absence of asthma or AERD.^65^

In Brazil, among the alternatives of biologics aimed at interrupting the action of IL-5, only Mepolizumab is indicated for CRSwNP.

## Indications

### Mepolizumab^46^

1 Severe eosinophilic asthma in adult and pediatric patients above 6-years of age.
2 Recurrent or refractory Eosinophilic Granulomatosis with Polyangiitis. (EGPA) as complementary treatment to corticosteroids in adult patients.
3 Hypereosinophilic Syndrome (HES) for patients aged 12 and older, lasting ≥6 months, without an identifiable secondary non-hematologic cause.
4 Severe Chronic Rhinosinusitis with Nasal Polyps (CRSwNP), as adjunctive therapy to intranasal corticosteroids in adult patients for whom systemic corticosteroid therapy and/or surgery have not provided adequate disease control.

### Benralizumab/Reslizumab^47^, ^48^

1 Severe asthma, as maintenance adjuvant treatment for asthma, with eosinophilic phenotype in adult patients (>18 years old).

## Posology and treatment duration

According to package insert recommendations, Mepolizumab^6^ is administered as a subcutaneous injection. For asthma, the recommended dosage is 100 mg every 4-weeks. In children aged 6–11 years, 40 mg should be administered once every 4 weeks. For EGPA, the dosage is 300 mg every 4-weeks. In CRSwNP, the dosage is 100 mg, administered once every 4-weeks.

Reslizumab is administered as an intravenous infusion every 4-weeks at a dosage of 3 mg/kg.^47^

Benralizumab is administered subcutaneously at a dosage of 30 mg every 4-weeks for the first three doses, followed by every 8-weeks thereafter.^48^

Little is known about the duration of use of biologics and the maintenance of clinical symptoms after treatment discontinuation. A recent study, evaluating the clinical follow-up of 134 patients with severe CRSwNP treated with Mepolizumab for 52-weeks, compared to a placebo group, showed that clinical improvement, quality of life, and corticosteroid use remained evident for up to 24-weeks after Mepolizumab discontinuation, a fact that should be considered by physicians.

## Safety

The safety and tolerance of anti-IL-5 agents have been established in studies for the treatment of asthma and CRS.^59^, ^60^ Anti-IL-5s are safe and well-tolerated, with the most common side effects being headache, injection site reactions, back pain, and fatigue.^59^ In a study with Mepolizumab in severe CRSwNP, the most observed side effects were pharyngitis, increased serum creatine phosphokinase and myalgias.^44^, ^56^ The use of Reslizumab in patients with severe CRSwNP was considered safe and well-tolerated. The side effects of Benralizumab include headache, pharyngitis, and injection site reactions.^61^

One concern related to the use of anti-IL-5 was the decrease in host defense.^56^ However, in clinical trials with Mepolizumab and Benralizumab used for 1-year, the frequency of upper respiratory tract infections was lower than that of the placebo group.^61^, ^62^

Regarding the possibility of an association between anti-IL-5 and the appearance of malignant tumors, it was observed that the incidence rate of malignancy was similar to that of the placebo group.^61^

Systemic reactions observed with the use of Mepolizumab and Benralizumab were hypersensitivity reactions in 2% and 1%–3%, respectively. Headache occurred more frequently during the use of Mepolizumab (20% higher compared to the placebo arm) and Benralizumab (7%–9%), also compared to the placebo arm (5%–7%).^61^

Recent RCTs and systematic reviews from the past 5-years confirm previous results regarding the adverse events/effects of IL-5-blocking biologics.^62^, ^63^, ^64^, ^65^

## Dupilumab (Anti-IL-4 and Anti-IL-13)

Dupilumab is a monoclonal antibody directed against the alpha subunit of the anti-IL-4 receptor (Anti-IL-4Ralfa), thereby blocking the action of IL-4 and IL-13. It was first approved for use in 2017 for moderate-to-severe atopic dermatitis: in March/2017 by the United States Food and Drug Administration (FDA)^66^ and in October/2017 by the European Medicines Agency (EMA).^67^

For the treatment of Chronic Rhinosinusitis with Nasal Polyps (CRSwNP), it was initially approved as an adjuvant treatment in adults lacking control of appropriate therapy: by the FDA in June/2019 and by EMA in September/2019. It was also approved for patient’s intolerant to systemic corticosteroids and/or unable to undergo surgical treatment. In Brazil, it has been authorized for use by ANVISA since 2017 for Atopic Dermatitis and since April 2020 for patients with asthma. Dupilumab was the first biological with a specific indication for CRSwNP authorized by major international regulatory agencies and in Brazil, with approval in July 2020.^68^

## Mechanism of action

IL-4 and IL-13 are potent mediators of type 2 inflammation, sharing the same receptor and signaling pathways. These cytokines are involved in IgE synthesis, eosinophil migration from blood vessels to inflamed tissue, mucus secretion, and airway remodeling. IL-4 is a key differentiation factor for the Th2 response, acting on T cells to differentiate into the Th2 subtype and inducing the production of type 2 cytokines and chemokines such as IL-5, IL-9, IL-13, TARC, and eotaxin. Additionally, IL-4 and IL-13 are responsible for B-cell isotype switching to IgE production.^2^

The evolution of the concept that inflammation in CRSwNP patients is mediated not only by Th2 lymphocytes but also by Innate type 2 Lymphoid Cells (ILC-2) highlighted the importance of targeting mechanisms that block inflammatory pathways beyond the classical Th2 pathway.^69^

Dupilumab is a recombinant human IgG4 monoclonal antibody directed against the Interleukin-4 Receptor alpha (IL-4Rα). Its blockage inhibits IL-4/IL-13 signaling, leading to a decrease in type 2 immune response.^70^

Dupilumab has a significant effect on local and systemic type 2 inflammatory biomarkers: it reduces eotaxin-3, Thymus and Activation-Regulated Chemokine (TARC/CCL17), periostin and total serum immunoglobulin E, IL-5, Eosinophil Cationic Protein (ECP), and leukotriene E4 in urine. The reduction in these biomarkers is similar or greater in subgroups with asthma and AERD, as evidenced in a post-hoc analysis of the SINUS-24 study.^71^

## Experience in other respiratory diseases

### Severe asthma

Several randomized, placebo-controlled clinical trials have been published involving patients with uncontrolled persistent asthma despite adequate treatment with inhaled corticosteroids and long-acting beta-agonists,^71^, ^72^ inhaled corticosteroids +1 or 2 rescue medications,^75^ or oral corticosteroids.^76^

These studies monitored the use of dupilumab for 12^72^ and 24^73^, ^74^, ^76^ weeks, and demonstrated a reduction in exacerbations, improved asthma control, enhanced pulmonary function, decreased oral corticosteroid use, improved quality of life, and increased productivity related to asthma. The doses varied between 200 and 300 mg subcutaneously, with application every two weeks proving more effective than every four weeks.^73^

Important subsequent publications have confirmed the efficacy and safety of this medication for asthma in both adults and children, such as QUEST, VENTURE, TRAVERSE, VOYAGE, and EXCURSION.^76^, ^77^, ^78^, ^79^, ^80^

## Efficacy in CRSwNP

Dupilumab is the first biological treatment approved for use in CRSwNP by the FDA, EMA, and ANVISA,^81^, ^82^ and since 2020, it has been approved for use in Sinonasal Polyposis regardless of the presence of asthma in Brazil.^68^

The first clinical trial evaluating Dupilumab in CRSwNP was conducted in 2016. Bachert et al.^83^ published a double-blind placebo-controlled clinical trial that randomized 60 adults with CRSwNP into two groups for 16-weeks: subcutaneous dupilumab (initial dose of 600 mg followed by 15-weekly doses of 300 mg) or corresponding placebo. Dupilumab promoted a significant improvement in quality of life, nasal obstruction, olfaction, nasal polyp size, tomographic scores, and asthma (clinical control and lung function).

In 2019, the results from the first randomized double-blind, multicenter, placebo-controlled clinical trials assessing the efficacy of Dupilumab added to standard treatment in adults with severe CRSwNP for 6 and 12 months were published^84^: the “LIBERTY NP SINUS-24” study randomized patients into two equal groups for 24-weeks, dupilumab 300 mg or placebo every two weeks; the “LIBERTY NP SINUS-52” study, into three equal groups for 52-weeks: a) 52-weeks with dupilumab 300 mg every two weeks, b) 24-weeks with dupilumab 300 mg every two weeks followed by 28-weeks with dupilumab 300 mg every four weeks, or c) 52-weeks with placebo every two weeks. Bachert et al. demonstrated, in both studies, a significant improvement in quality of life, nasal obstruction, polyp size, nasal endoscopy, and lung function, regardless of whether the patient had undergone prior endoscopic sinus surgery.^84^, ^85^

Dupilumab also led to a reduction in the concentrations of eosinophilic inflammation biomarkers: serum IgE, eotaxin-3, periostin, and TARC; tissue IgE, eosinophilic cationic protein, eotaxin-2, PARC, IL-13, periostin, and IL-5.^86^, ^87^ However, the diagnosis of Allergic Rhinitis or the number of eosinophils in the serum does not interfere with the intensity of the response to Dupilumab, the frequency of systemic corticosteroid use, or the indication for sinonasal surgery.^88^, ^89^, ^90^

Several post-hoc analyses of these studies were published,^83^, ^91^, ^92^, ^93^ and showed improvement in quality of life, nasal congestion/obstruction, reduced need for surgery and oral corticosteroid use, polyp size, tomographic appearance, lung function, and olfaction, the latter irrespective of prior sinusectomy.^90^ Chong et al. endorsed these results through a systematic review.^93^ There is proven beneficial effect in CRSwNP associated with Aspirin-Exacerbated Respiratory Disease (AERD)^92^, ^94^ and in Allergic Fungal Rhinosinusitis (AFRS).^95^ The relative risk for reoperation after starting dupilumab use reduces considerably.^85^, ^93^ When associated with intranasal corticosteroids, it reduces sick leave days and improves work productivity^93^ (Table 3).

**Table 3:** Clinical trials on the efficacy of Dupilumab in CRSwNP.

Study	Method	Participants	Intervention	Outcomes/Results
Bachert (2019)^83^	DBPCCT	CRSwNP	SINUS-24 (24 weeks):	Significant improvement in both studies compared to the placebo:
		SINUS-24: 276 patients	• Dupilumab (300 mg 15/15d) (n = 143)	• SNOT-22
			• Placebo 15/15 d (n = 133)	• Rhinosinusitis disease severity (VAS)
		SINUS-52: 448 patients	SINUS-52: (52 weeks):	• Nasal congestion
			• Dupilumab (300 mg 15/15 d) (n = 150)	• UPSIT
			• Dupilumab (300 mg 15/15d for 24	• Nasal polyps
			• Weeks, and 30/30 d for 28 weeks (n = 145)	• Nasal endoscopy (Lund-Kennedy)
			• Placebo 15/15 d (n = 153)	• ACQ5
			Both studies:	• Pulmonary function (FEV1)
			Mometasone 100 μg/nostril 12/12 h for 4 weeks before and during the study.	
Bachert (2016)^84^	DBPCCT	60 patients with CRSwNP refractory to INC	Dupilumab (starting with 600 mg, 300 mg per week) for 16 weeks (n = 30)	Significant improvement:
			Placebo for 16 weeks (n = 30)	• SNOT-22
			Mometasone 100 μg/nostril 12/12 h for 4 weeks before and during the study.	• Rhinosinusitis severity (VAS)
				• Nasal congestion
				• Olfaction (UPSIT)
				• Nasal polyps score (NPS)
				• CT (LMS)
				• ACQ6
				• FEV1

CRSwNP, Chronic Rhinosinustis with Nasal Polyps; DBPCCT, Double-Blind randomized Placebo-Controlled Clinical Trial; SNOT-22, SinoNasal Outcomes Test-22; VAS, Visual Analog Scale; UPSIT, University of Pennsylvania Smell Identification Test; ACQ, Asthma Control Questionnaire; NPS, Nasal Polyps Score; LMS, Lund-Mackay Score; FEV1, Forced Expiratory Volume in 1 s; INC, Intranasal Corticosteroid.

There is a rapid and sustained improvement in olfaction with dupilumab, relieving a important symptom in severe CRSwNP. This post-hoc analysis of the SINUS-24 and SINUS-52 studies revealed a rapid improvement (as of day 3) in patient-reported olfaction and the University of Pennsylvania Smell Identification Test (UPSIT), which progressively improved over the study periods. The improvements were not affected by the duration of CRSwNP, prior FESS, or coexisting asthma and/or respiratory diseases exacerbated by nonsteroidal anti-inflammatory drugs. The improvement in initial olfaction scores correlated with all measured local and systemic type 2 inflammatory markers, except for total serum immunoglobulin E. The proportion of anosmic patients in the dupilumab group decreased from 78% to 28%.^94^, ^95^, ^96^

A variety of real-life studies have highlighted the subsequent impact of dupilumab.^97^, ^98^ One multicenter observational Phase IV real-life study (DUPIREAL) in patients with uncontrolled severe CRSwNP (n = 648) showed a moderate or excellent response in 96.9% of patients at 12-months based on the EPOS 2020 criteria: significant improvement in polyp size, quality of life assessed by SNOT-22, and olfaction by “Sniffin' Sticks” over 12-months. Patients with prior surgery or asthma also experienced faster improvements in parameters; however, these differences were not statistically significant.^98^

A comparative analysis between endoscopic surgery vs. biologicals revealed that FESS resulted in improvements comparable to dupilumab: in quality of life by SNOT-22 after 24 and 52 weeks; in olfaction identification at 24-weeks. Compared to another biological, omalizumab, FESS promoted superior improvements in quality of life according to SNOT-22. However, FESS provides significantly greater reductions in polyp size compared to therapies with omalizumab, dupilumab, and mepolizumab.^99^ Hopkins et al. evaluated the efficacy of dupilumab in patients with prior surgery and found that the drug resulted in improvements in patients with CRSwNP and reduced the use of oral corticosteroids and the need for new surgeries, regardless of surgical history, with greater magnitude in endoscopic results for patients with a shorter interval between the last surgery and the start of medication. Dupilumab promoted significant improvements in all subgroups divided by the number of surgeries and the time since the last surgery. The best results regarding nasal polyp size and sinus computed tomography were in patients who underwent the last surgery <3-years ago compared to patients who had surgery ≥5-years ago.^100^

However, due to the high cost of biologicals, a Markov decision tree economic model cohort-style study conducted in the USA showed lower cost-effectiveness of dupilumab (SINUS-24/SINUS-52) versus a cohort of patients who underwent FESS (considering surgery costs in that country). Nevertheless, a more extensive analysis of the costs involved is necessary, as this study may have underestimated some expenses related to surgery, revision surgery rates, as well as indirect costs associated with absenteeism, presenteeism, and treatment of coexisting asthma.^101^

A network analysis study (7 clinical trials, n = 1913) was conducted aiming at comparing the efficacy and safety of different biologicals in the treatment of CRSwNP, as there are currently no comparative studies among them. Primary outcomes were polyp grade, nasal congestion, and severe adverse events; secondary outcomes included quality of life by SNOT-22, olfaction severity, UPSIT, and Lund-Mackay. Dupilumab showed better effects in reducing sinonasal polyps and the severity of nasal congestion.^102^

The best way to decide the choice of treatment is based on double-blind, placebo-controlled randomized clinical trials. The EVEREST study (NCT04998604) is currently in recruitment, representing the first comparative study between two biologicals for severe CRSwNP and asthma. Patient recruitment began in September/2023, and the results will provide evidence to help choose between dupilumab vs. omalizumab in severe CRSwNP and asthma.^103^

## Efficacy in patients with CRSwNP and AERD

Patients with AERD represent a subgroup of CRSwNP with a much more severe phenotype of sinonasal disease associated with repeated surgeries. Results related to AERD (NSAID-exacerbated respiratory disease/aspirin-exacerbated respiratory disease) were evaluated in a post-hoc analysis that compiled data from the SINUS-24/SINUS-52 studies on the efficacy and safety of dupilumab in AERD versus NSAID-tolerant CRSwNP. In patients with severe uncontrolled CRSwNP, dupilumab more significantly improved objective measures and patient-reported symptoms in the presence of AERD than in their absence, and it was well-tolerated in both patient groups.^104^ Another post-hoc analysis showed that dupilumab not only significantly improved clinical scores related to CRSwNP on week 24, but also improved lung function and asthma control and decreased urinary LTE4 levels. Inhibition of IL-4 and IL-13 may reduce LTE4 production, which could explain the greater reduction in urinary LTE4.^105^

Anti-IL-4/13 and anti-IgE appear to be more effective than anti-eosinophilic therapies in patients with AERD.^106^ A prospective pilot study evaluated whether biologicals could induce NSAID tolerance in patients with AERD similarly to desensitization. The study randomized the use of benralizumab, dupilumab, mepolizumab, or omalizumab in individuals with severe asthma and type 2 inflammation, i.e., high levels of total IgE, atopy, and eosinophils. After 6-months using the biological, NSAID tolerance was confirmed by an oral provocation test with greater frequency of omalizumab and dupilumab, indicating that the use of these therapies could induce aspirin tolerance in a larger proportion of patients with AERD than anti-eosinophilic biological action.^106^

A real-life retrospective study involving 74 individuals who received biologicals for AERD reported that patients who received dupilumab benefited from a significant reduction in SNOT-22 quality of life scores and reduced use of oral corticosteroids and antibiotics, whereas anti-IgE or anti-IL-5/IL-5Rα showed no statistically significant results.^107^

A network meta-analysis showed that dupilumab was among the most beneficial of 8 treatments (29 clinical trials, n = 53,461) with biologicals for patients with AERD The study showed that dupilumab promoted improvements in: a) Sinusitis symptoms; b) Quality of life by SNOT-22; c) Olfaction; d) Reduction in the frequency of oral corticosteroid use; e) Rescue surgeries; f) Polyp size, and g) The Lund-Mackay CT score. Comparisons between biologicals showed that dupilumab is among the most beneficial for 7 out of 7 outcomes, omalizumab for 2 out of 7, mepolizumab for 1 out of 7, and desensitization for 1 out of 7.^108^

Real-life studies are an important tool to strengthen evidence on the actual impact of medications on patients with severe CRSwNP and to fill in gaps in the efficacy and efficiency data of Dupilumab in severe CRSwNP. A large, long-term, prospective global registry, AROMA, has been initiated to better understand the utilization, efficacy, and safety of dupilumab for the treatment of CRSwNP in real-life clinical practice. The evaluations will consist of objective measures and various patient-reported questionnaires, treatment patterns, concomitant medications, long-term safety, and progression of CRSwNP, including those with coexisting diseases.^109^

## Indications

Dupilumab is indicated as adjuvant treatment in CRSwNP in adults who have failed previous treatments, or who are intolerant or contraindicated to oral corticosteroids and/or surgery. Its indication follows the criteria previously described for the use of biologicals.

It should not be used to treat patients with acute bronchospasm or status asthmaticus, or patients with helminthic infections. These three groups of patients should be treated prior to the initiation of Dupilumab therapy.^68^

Despite not being described in the package insert, an exploratory study found that the use of FESS combined with the biological was better than the use of the biological alone and resulted in a significant and sustained decrease in polyp burden compared to biological therapy alone. Patients underwent surgery approximately 30-days after starting biological therapy and were followed up for 12-months. The reduction in the impact of polyposis was greater in the group submitted to surgery (4.73 to 0.09 vs. 5.22 to 3.38).^110^ Future studies may confirm this result in a larger sample of patients.

## Posology

The dosage of dupilumab for CRSwNP is administered subcutaneously. There is no loading dose in patients with CRSwNP, unlike atopic dermatitis or asthma. The dose is 300 mg, which is generally administered for the first time in the medical practice to train and empower the patient on how to self-administer the medication, and, subsequently, at home. It can be self-administered by the patient, administered by a healthcare professional, or by a caregiver. Afterward, every 2-weeks, 300 mg should be administered subcutaneously. If the patient forgets to administer a dose, it should be carried out as soon as possible. After that, the regular dosing regimen should be resumed.^88^

One of the major concerns with the use of biologicals is how the treatment frequency will be after the first 12-months. A prospective observational real-life cohort study (n = 228) showed the possibility increasing the interval between dupilumab doses in CRSwNP. There was a 2-year follow-up for increasing the dose interval to every 24-weeks. Patients showed improvement in all outcomes at 48 and 96 weeks: nasal polyp score, quality of life by SNOT-22, olfaction by “Sniffin' Stiks”, and improvement in the Asthma Control Test. There were no significant changes in individual coprimary outcomes from 24 weeks onwards.^111^

## Safety

Although there is a concern regarding the induction of conjunctivitis by dupilumab, a systematic review showed that this side effect was associated with studies on atopic dermatitis but not in patients with asthma or CRSwNP.^49^ More common adverse events were more frequent with the placebo, such as nasopharyngitis, worsening of nasal polyps and asthma, headache, epistaxis, and erythema at the injection site.^112^

In November/2023, the first large-scale comparative analysis of adverse event profiles of dupilumab (112,560 adverse events), omalizumab (24,428 adverse events), and mepolizumab (18,741 adverse events) was published, endorsing a strong relationship between dupilumab and ophthalmological adverse events, such as blurred vision (ROR = 3.80) and visual impairment (ROR = 1.98). Dupilumab was the only biological associated with injection site reactions (ROR = 8.17). However, omalizumab presented the strongest association with anaphylaxis (ROR = 20.80). This reinforces the need to inform the patient about the possibility of these reactions with these biologicals so that they can communicate with their attending physician if they occur, to be safely managed.^113^

Use during pregnancy category: B. This medication should not be used by pregnant or lactating women without medical guidance. Safety and efficacy in pediatric patients under 18-years old have not been established for CRSwNP.^112^

It is indicated for the treatment of children aged 6-months to 11-years old with severe atopic dermatitis, whose disease is not adequately controlled with topical treatments or when these treatments are not advised.^112^

## Other anti-IL-13

Tralokinumab and lebrikizumab are two other anti-IL-13 antibodies that have not been studied in patients with CRS; however, there are some studies involving the tralokinumab biological in patients with asthma.^113^

## Future biologics for chronic eosinophilic rhinosinusitis

While current biologics focus on the adaptive type 2 immune response (primarily cytokines such as IL-5, IL-4, and IL-13, in addition to IgE), new immunobiologicals have been developed targeting the innate immune response. This evolution paralleled the understanding of CRS pathophysiology: adaptive immune mechanisms related to CRS were unveiled in the mid-1990s, while innate immunity-related mechanisms were revealed only 10-years later.

Among the cytokines of innate immunity with potential as therapeutic targets, two stand out in the literature: IL-33 and TSLP. Both of them are produced in the epithelium and have a broader capacity to induce eosinophilic response, stimulating both type 2 lymphocytes (Th2) and innate lymphoid cells.

Regarding anti-IL-33 immunobiologicals, two different drugs were evaluated for safety ‒ Phase II trials (Clinicaltrials.gov Identifier: NCT02743871 and NCT02170337), while a third concluded a Phase III trial with a drug called etokimabe (Clinicaltrials.gov Identifier: NCT03614923). In this trial, whose data were only published on ClinicalTrials.gov, etokimabe, when associated with mometasone furoate spray, did not show superiority over mometasone associated with placebo, either in reducing polyps (assessed by the Nasal Polyp score ‒ NPS) or in improving quality of life (assessed by SNOT-22). However, it should be noted that the number of evaluated patients was only 105 individuals. Four anti-TSLP drugs are currently in Phase III trials according to Clinicaltrials.gov (NCT04851964, NCT05324137, NCT05891483, and NCT06036927), with Tezepelumab being the first andmost studied. Tezepelumab was initially evaluated for asthma, with promising results, reducing the number of exacerbations throughout the year.^114^, ^115^, ^116^, ^117^ Interestingly, the results were not correlated with the participants' serum eosinophil count^115^ and were equally effective in participants with associated allergic rhinitis (either seasonal or perennial).^118^

Current review articles, including key clinical trials conducted so far, emphasize not only the reduction in exacerbations but also improvements in asthma-related quality of life questionnaires, as well as reduced Forced Expiratory Volume in 1 s, in L (FEV1), Fractional Exhaled Nitric Oxide (FeNO), serum eosinophils, and total serum IgE.^119^, ^120^, ^121^, ^122^, ^123^

Although the results of research with CRSwNP have not yet been officially disclosed, some asthma studies included participants with associated CRSwNP, allowing for post-hoc analyses.

Emson et al.^124^ conducted a post-hoc analysis of the results of the PATHWAY trial (Clinicaltrial.gov Identifier NCT02054130), during which the participants received subcutaneous tezepelumab at different doses every 2 or 4-weeks or a placebo for 52-weeks. Interestingly, the administration of 210 mg of tezepelumab every 4-weeks reduced asthma exacerbations, both in patients with or without CRSwNP.

Laidlaw et al.^125^ performed a post-hoc study of the NAVIGATOR trial (Clinicaltrials.gov Identifier NCT03347279), which included 1059 participants with asthma, 118 of whom had associated CRSwNP. Of these, 62 received 210 mg of tezepelumab every 4-weeks, while 56 were given placebo. Patients who received tezepelumab showed a significantly greater reduction in SNOT-22 than those who received placebo, either after 28 or 52 weeks of use (mean difference between treatments of 12.57 points on week 28 and 10.58 points on week 52).

Although the studies mentioned above suggest that tezepelumab may have an impact on CRSwNP, larger studies including objective assessments are needed. The results of studies NCT04851964, NCT05324137, NCT05891483, and NCT06036927 should provide a clearer understanding of tezepelumab’s role in CRSwNP.

## Future biologics for non-eosinophilic chronic rhinosinusitis

Patients with refractory chronic rhinosinusitis without nasal polyps to topical corticosteroid treatment and surgical interventions still lack an alternative treatment option with the use of immunobiologicals.

The pathophysiology of CRS without polyps is less understood, likely due to fewer patients having corticosteroid-refractory disease in these cases. Some recent studies suggest that this refractoriness may be related to T2 disease (eosinophilic), even in the absence of nasal polyps.^126^, ^127^ Other authors also mention that current immunobiologicals (especially dupilumab and tezepelumab) act not only on the T2 response, but also on epithelial barrier dysfunction.^128^, ^129^

Other ongoing clinical trials (Clinicaltrials.gov Identifier NCT 04678856, NCT04430179, and NCT04362501) are evaluating the potential effect of dupilumab and other anti-T2 biologics in CRSsNP, and which subtypes could benefit from this medication.

## Biologicals for secondary CRS

### Eosinophilic granulomatosis with polyangiitis (EGPA)

So far, mepolizumab (anti-IL-5) is the only biologic authorized by various regulatory agencies (FDA, EMA, and ANVISA) for use in patients with EGPA, at a dose of 300 mg every 4-weeks, with usage restricted to specific situations, depending on the stage or severity of the disease.^130^ According to the American College of Rheumatology,^130^ mepolizumab should not be used as monotherapy or combined with immunosuppressive agents in severe cases of EGPA. In non-severe cases, mepolizumab may be considered in combination with corticosteroids to induce remission. For patients who have achieved disease remission and require maintenance doses, mepolizumab is not recommended if the initial presentation of the disease was severe. In cases of EGPA relapse during the immunosuppressive therapy phase with corticosteroids or methotrexate, the American College of Rheumatology recommends adding mepolizumab to the current therapy instead of adding another immunosuppressive or switching to another biologic. Despite mepolizumab being the most tested drug for patients with EGPA, there are still numerous uncertainties regarding the optimized dose, frequency, and the best clinical profiles that can predict therapeutic responses of EGPA patients to mepolizumab.^131^

Other agents, such as reslizumab (anti-circulating-IL-5) and benralizumab (anti-IL-5 receptor), have also been tested for patients with EGPA. Currently, a randomized, double-blind study is underway, comparing head-to-head benralizumab with mepolizumab for patients with refractory or recurrent EGPA, primarily evaluating the percentage of patients in remission on weeks 36 and 48 of treatment (ClinicalTrials.gov Identifier: NCT04157348).^132^

In general, these medications can lead to mild-to-moderate adverse events, with the most common comprising headaches, local reactions, back pain, fatigue, rhinorrhea, and nasal congestion. More severe adverse events are rare and include anaphylaxis.

### Granulomatosis with polyangiitis (GPA)

Rituximab (anti-CD20 antibody) is the most extensively tested biologic for patients with GPA, previously known as Wegener's Granulomatosis. Several studies have demonstrated the benefits of rituximab in patients with GPA in different phases of the disease, whether in induction of remission, the maintenance phase, relapse control, or in forms refractory to conventional treatments with cyclophosphamide.^133^, ^134^, ^135^ However, most of these studies present low levels of evidence with small case series,^135^, ^136^ so the main treatment guidelines for ANCA-associated vasculitis, including GPA, are quite heterogeneous regarding the doses and recommendations for the use of rituximab in different phases of the disease.^130^, ^137^, ^138^, ^139^

An additional consideration is the risk of adverse events, including severe infections, which are more frequent with this biologic than with the anti-T2 agents mentioned earlier.

### Olfaction and biologicals

In this section, we will outline the updates associated with the effect of biologicals on the olfaction of patients with CRSwNP since the last edition of these Guidelines.^140^ In addition, we will discuss the criteria related to this sense, both for the prescription of these drugs and for the continuation of their use after the start of treatment.

Among the most promising effects of biologics in patients with CRSwNP is their impact on olfaction. Studies using validated psychophysical tests to assess the sense of smell report clinically significant improvements in patients with anosmia, mild hyposmia, or even normosmia after treatment with biologics. It is important to highlight that anosmia is one of the five criteria established by the EPOS 2020 for the indication of CRSwNP treatment with biologics.^2^ The diagnosis of anosmia must be made through a validated psychophysical test, not solely on questionnaires assessing the patient’s perception of olfactory loss. Subjective quantification of olfactory loss by the patient is less reliable and reproducible, and a high percentage of patients are unaware of any olfactory loss.^141^, ^142^, ^143^ Psychophysical tests are not considered objective evaluation methods, as they depend on the patient’s response, but they do present less subjectivity compared to questionnaires and assess variations in olfactory capacity over time with greater sensitivity.

In Brazil, although not yet widely used in most otolaryngology practices, validated olfaction tests have become more accessible and increasingly widespread. The most commonly used tests are the University of Pennsylvania Smell Identification Test (UPSIT)^144^ and the Connecticut Chemosensory Clinical Research Center Test (CCCRC).^145^ For a patient to meet the olfactory loss criterion for biologic prescription, they are required to be classified as anosmic, regardless of the applied test (Table 4). Cases of mild-to-moderate hyposmia tend to resolve well with the use of topical and systemic corticosteroid therapy.^146^

**Table 4:** Classification of the degrees of olfactory loss according to the UPSIT, CCCRC, and MULTISCENT-20 tests.

Categories of olfaction	UPSIT	CCCRC	MULTISCENT-20
Normosmia	Women: > 34	6 to 7	≥15
	Men: > 31		
Mild to severe hyposmia	Women: 17‒31	2 to 5.75	11 to 14
	Men: 19‒34		
Anosmia	Women: < 17	0 to 1.75	≤10
	Men: < 19		

UPSIT, University of Pennsylvania Smell Identification Test; CCCRC, Connecticut Chemosensory Clinical Research Center Test; MULTISCENT-20, Multiscent-20 Digital Odor Identification Test.

Among the biologicals available for the treatment of patients with CRS in Brazil, dupilumab (IL-4 and IL-13 inhibitor) is the alternative that has shown the greatest efficacy in improving olfactory function. In a randomized, double-blind, controlled clinical trial, patients treated with dupilumab for 16-weeks, combined with topical mometasone furoate, showed a 14-point increase in the UPSIT test, compared to the control group (placebo plus the same nasal spray), which showed no improvement.^108^ This difference represents more than three times the value perceived as clinically relevant by the patient,^147^, ^148^ resulting, in some cases, in the recovery to normal levels of olfaction. The robustness of these results was corroborated by Phase III trials and the sub-analyses of these studies, which revealed a significant increase in olfactory function after 24-weeks, although with a difference of 10.5 points between groups, regardless of confounding factors such as disease duration, previous surgeries, and concomitant respiratory conditions.^109^, ^149^ The baseline olfactory scores correlated with all measured local and systemic type 2 inflammatory markers, except for total serum immunoglobulin E. Real-life studies also reinforce the positive impact of dupilumab on olfaction. Significant improvement in olfactory function, even in patients with persistent polyps, was observed in a cohort of 53 patients using dupilumab.^150^ Additionally, this biologic significantly improved the patients’ olfactory function, regardless of the number of previous nasal surgeries and the time elapsed since the last surgery.^125^

Omalizumab, an IgE inhibitor, also promoted improvement in olfaction in two Phase III clinical trials, although less pronounced than dupilumab. In both studies, a statistically significant difference of 3.8 points on the UPSIT test was observed between groups. However, this change was not perceived when evaluating patients with the self-report olfactory assessment scale.^33^ The difference persisted for up to 52-weeks, but treatment discontinuation resulted in subsequent deterioration in olfactory function.

Mepolizumab, an anti-IL-5 monoclonal antibody, demonstrated, in comparative studies with placebo, a modest improvement in olfactory function assessed by visual analog scales. However, there was no significant improvement in olfactory function measured by psychophysical tests.^44^, ^59^, ^60^

In spite of the lack of direct studies between these biological agents, innovative data comparison methods, such as network meta-analysis and real-life studies, also suggest the superiority of dupilumab over omalizumab, dupilumab and benralizumab in improving olfactory function.^132^, ^151^, ^152^

## How to carry out olfactory tests already validated for Brazil?

### University of Pennsylvania Smell Identification Test (UPSIT)

**Figure 2:**
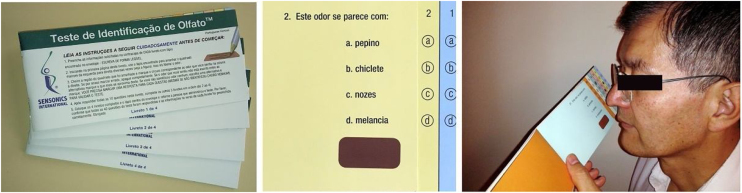
The four booklets of the University of Pennsylvania Smell Identification Test (UPSIT), one of the test pages, and a patient attempting to identify one of the odors after scraping the strip with the booklet close but without touching their nose.

The UPSIT test consists of 40 odors distributed across 4 booklets with 10 pages each. The patient scrapes the brown strip at the bottom of the page to release the odor, places the booklet within 1.0 cm of their nose, and is required to choose one answer among four possible alternatives (Fig. 2). The responses can be recorded directly on the test or on a separate sheet for later correction. The number of correctly identified odors constitutes the score on the test, and olfactory function can be classified as normal or altered according to Table 4.

### Connecticut Chemosensory Clinical Research Center Test (CCCRC)

The CCCRC tests both olfactory threshold and the identification of different odors, allowing for both the quantitative and qualitative assessment of olfaction. Moreover, it independently investigates each nasal cavity, enabling the discrimination of olfactory alterations on either side and presenting a low production cost.

In order to conduct the olfactory threshold test, seven concentrations of Butanol (n-butyl alcohol) are used, packaged in identical and numbered vials from 1 to 7, with the highest to the lowest concentration. A vial containing distilled odorless water (vial 8) serves as a control. The olfactory threshold is determined by presenting the individual with two identical vials, one containing distilled water and the other a butanol solution. The test begins by comparing the lowest concentration of butanol (vial 7) with distilled water (vial 8). If the patient is able to differentiate the two substances, the olfactory threshold score in the tested nostril corresponds to the number of the butanol vial (in this case, 7). On the other hand, if the individual cannot identify the odor, vials with more concentrated butanol solutions are consecutively presented, alternating with the distilled water vial. If the patient cannot identify even the most concentrated butanol solution (vial 1), the final score is 0. The same steps should be repeated for the contralateral nasal cavity.

As for the substance identification test, eight substances are used: coffee powder, cinnamon powder, baby powder, *paçoca* (peanut candy), cocoa powder, neutral soap, and naphthalene. The substances are placed in opaque vials, preventing visual identification of the content. Individuals are provided a list beforehand containing the names of the eight substances in the test, along with the names of eight distractor substances. For each presented vial, the individual must name one of the substances from the list. After testing the 8 substances in one nostril, the same steps are repeated in the contralateral nostril, presenting the vials randomly to avoid memorization of the order. The trigeminal nerve function is evaluated at the end of the test with the presentation of menthol (Vick®), but the identification of this substance is not included in the final score. The identification test score is obtained by scoring from 0 to 7 for each nasal cavity, depending on the number of correctly identified odors.

The final test score is independently calculated for each nostril by averaging the olfactory threshold value and the identification test, and ranges from 0 to 7 points.

## MULTISCENT-20 digital odor identification test

**Figure 3:**
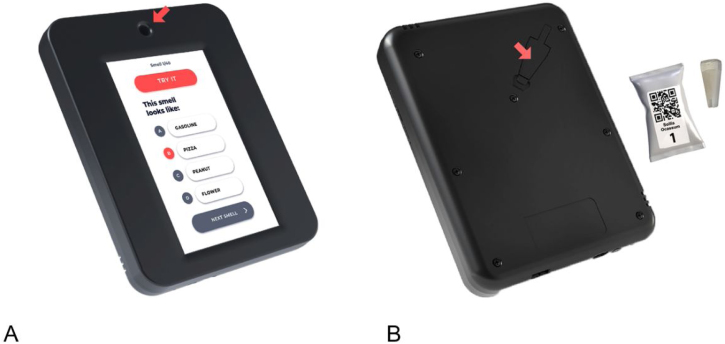
(A) Front view of the Noar MultiScent-20® digital device. It is a 7-inch touchscreen tablet used to digitally demonstrate aromas. The odor release opening is indicated by an arrow. (B) Rear view of the device. Capsule with individual odor storage. The capsules are loaded through an insertion port (arrow) on the back of the device.

The Multiscent-20 is a tablet that features an odor storage and release system. It is a portable hardware device with a touchscreen, integrated with a microcapsule system capable of presenting up to 20 different odors through a flow of dry air released at the odor dispenser opening (Fig. 3A). The capsules are loaded through an insertion port on the back of the device (Fig. 3B). The software application presents, controls, and records responses in an automated manner.^153^

The digital odor identification test utilizes the mandatory response paradigm among four alternatives. The olfactory assessment begins with the individual receiving instructions presented by an “avatar”, as described below:

1 This assessment consists of a test containing 20 odors.
2 Sit in a comfortable position, and when it’s time to trigger the odor, hold the device 10 cm away from your face.
3 The next screen will display the phrase “THIS SMELL IS SIMILAR TO” with four response options. READ ALL OPTIONS BEFORE pressing the “RELEASE SMELL” button.
4 By pressing the “RELEASE SMELL” button, a small opening at the top frontal portion of the device will release the odor for 5 s. You can press the “RELEASE SMELL” button up to two times.
5 After sensing the odor, select the option corresponding to the odor you smelled and press the “NEXT” button to proceed. If the odor you sensed is not among the alternatives, select the option that is the closest match.
6 The number of correct answers will be revealed after the test is completed.

The number of correct responses corresponds to the odor identification test score. Olfactory function is classified as normosmia (≥15 points), hyposmia (14 to 11 points), and anosmia (≤10 points) (Table 5).

**Table 5:** Eligibility questionnaire.

Variables	Score
*Sub-item ‒ Symptom severity*^154^	
a) SNOT-22 (validated for Brazilian Portuguese)	
< 20	0
20‒50	1
>50	2
b) VAS for nasal obstruction/congestion or rhinorrhea (consider the worst)	
< 3	0
3‒7	1
> 7	2
c) Olfactory Test (UPSIT/Connecticut)	
< 3 (normosmia or mild hyposmia)	0
3‒7 (moderate hyposmia)	1
> 7 (severe hyposmia or anosmia)	2
d) Number of previous surgeries	
0	0
1	1
2	2
≥3 or contraindication for undergoing surgery	3
e) Use of systemic CS/year	
0	0
1 or 2	1
> 2	2
*Sub-item ‒ Extent of the disease*	
f) Polyp Size (Nasal Polyp Score ‒ bilateral)	
0	0
1‒2	1
3‒4	2
≥ 5	3
g) Sinus opacification (Lund-Mackay – bilateral)	
0‒4	0
5‒8	1
9‒16	2
>16	3
*Sub-item ‒ Comorbidities*	
h) Asthma	
No	0
Mild	1
Moderate/severe	2
i) NSAID Intolerance	
No	0
Yes	2
*Sub-item ‒ Biomarkers*	
j) Serum eosinophilia	
< 150	0
150‒300	1
> 300	2
k) Tissue eosinophilia	
< 10	0
10‒43	1
> 43	2

## Criteria for indicating biologics in CRSwNP with type 2 inflammation

The therapeutic decision-making process for cases of CRSwNP previously indicated surgery after the failure of clinical treatment. However, with the emergence of biologics for the treatment of CRSwNP and the growing evidence of their efficacy and safety in managing this disease, a new decision-making flow needed to be outlined: that, with the failure of initial clinical treatment and appropriate surgery, there might be an indication for biologics. This approach is based on considerations related to safety and efficacy, as well as cost-effectiveness.

Given the rapid advancement of knowledge in this field, it is imperative to regularly update the guidelines for biologics eligibility criteria tailored to the local context, considering the regional characteristics of the disease, resource availability, and the funding of the healthcare system. Faced with the diverse realities around the world, certain guidelines may not be directly applicable in other regions, a fact that may pose challenges and create confusion for medical teams and national regulatory agencies in the indication and funding of these treatments.

In an effort to mitigate this issue, we propose a new eligibility criterion for biologics for patients with CRSwNP, based on four pillars of indication:

1 Impact of the disease on the patient’s life, whether in the presence of specific symptoms or in overall quality of life;
2 Extent of sinonasal disease;
3 Presence of type 2 comorbidities, such as asthma or AERD;
4 Presence of biomarkers to define type 2 inflammation.

In order to consider the use of a biologic, it is mandatory for the patient to present the following conditions: 1) CRSwNP; 2) Appropriate priorsinonasal surgery or absolute surgical contraindication.

Once the presence of the mandatory conditions is confirmed, the eligibility questionnaire below should be applied, which consists of 11 variables, with specific scores for each, resulting in a final score ranging from 0 to 25. All items below must be assessed: A final score <14 is highly suggestive of NOT RECOMMENDING the use of biologics, while patients with scores ≥14 are INDICATED for biologic use. It is important to emphasize that the higher the score, the greater the severity of the disease, with a higher probability of benefit from biologics, and, therefore, a stronger indication.

This scoring system was based on a pilot evaluation conducted by the authors, including 58 patients (29 considered not recommended for biologic use and 29 with an indication). The questionnaire proved to be highly sensitive in discriminating the “not recommended” and “indication” groups, considering a cutoff score of 14 points (Cronbach’s alpha 0.84; ROC curve with AUC 0.9828, sensitivity of 0.96, and specificity of 0.93 ‒ data not published).

We perceive two major advantages in this innovative and pioneering method on a global scale. First, it encompasses a comprehensive assessment of patients by incorporating all relevant clinical and laboratory factors related to this disease. Second, it is an evaluation that is easily adaptable to the rapid advancements in the understanding of the disease and potential changes in cost-effectiveness, where specific scores and cutoff points can be readily adjusted without the need to overhaul the entire evaluation scheme.

## Evaluation of the clinical response to the biologic ‒ disease control

When a biologic is selected to treat uncontrolled severe CRSwNP, it is important to monitor the patient's response to the medication. In order to avoid inappropriate treatment and unnecessary costs, the response to treatment should be reassessed every 6-months. To this end, the following criteria should be considered: VAS for nasal obstruction and/or SNOT-22, oral corticosteroid use, EPN by endoscopy, olfactometry, and asthma control (Table 6).

**Table 6:** Assessment of clinical response to the biologic.

Improvement in quality of life (SNOT-22): ≥ 9-points and/or VAS: ≤ points in nasal obstruction
Reduction in oral corticosteroid use (up to 1 cycle per semester)
Improvement in olfaction (UPSIT/Connecticut): By at least 1 degree
Reduction in nasal polyps (NPS): Decrease ≥ 2 points
Asthma control: Absence of exacerbations
Types of responses:
4‒5: Good
1‒3: Moderate
None: Non-responder

If the patient shows improvement in at least 4 of the 5 criteria, the response is considered good. Achieving a response in up to 3 criteria is considered moderate, and treatment should be maintained in both cases. However, when none of the criteria are met, the patient is considered a non-responder, and the option is to switch to another biologic or undergo a new surgery.

## Disease remission

Remission of a disease is defined as complete control of symptoms with objective markers. The recent application of this concept to the management of inflammatory airway diseases has promoted the concept of clinical remission, using the treat to target approach.^155^ The idea of remission is well established in rheumatology and gastroenterology, and is emerging in the field of airway diseases. In asthma treatment, the concept of remission is considered as the absence of exacerbations, stabilization of symptoms, and the possibility of normalizing inflammatory markers, which indirectly reflect lung function and inflammation.^156^ There is still no definitive definition for remission in CRSwNP, as in other specialties. In gastroenterology, for example, digestive endoscopy is used as a criterion that documents the recovery of the epithelium and mucosa.

Thus, expanding this concept to CRSwNP, clinical remission would be considered the normalization of symptoms and signs (SNOT-22, VAS, olfaction, EPN) through nasal endoscopy demonstrating normal sinonasal mucosa, with the maintenance of treatment.

Defining the criteria and the concept of remission in CRSwNP will allow for the identification of favorable patient outcomes. New research with more accurate biomarkers will be necessary to properly stratify these patients.

## Participatory therapeutic plan

The involvement of patients in therapeutic decision-making has always been integral to well-practiced medicine. Precision medicine involves tailoring the treatment for chronic diseases of the paranasal sinuses to each individual. With the evolution of our understanding of available therapeutic alternatives, it becomes crucial for the physician to possess the ability to convey, through suitable and practical language, the risks, and benefits of different options to their patients.

Advancements in medicine have not yet enabled the prevention or definitive cure of CRS. In practice, the goal is to minimize the significant impact of the disease on the patients’ quality of life.

The introduction of biologics, supported by scientific and real-life evidence demonstrating disease control in patients deemed severe and uncontrolled, and without the adverse effects associated with oral corticosteroid use, has marked a pivotal moment in the management of these patients.^2^ This ongoing evolution reinforces the importance of consistently sharing realistic expectations and updated therapeutic options.

Participatory treatment embodies the practical application of precision medicine, where patients may share phenotypic similarities but very diverse endotypes. The primary challenge lies in selecting and prioritizing therapeutic options within the constraints of finite and scarce financial resources. The definitive solution to this ongoing dilemma hinges on acquiring objective cost-effectiveness information, which is currently limited. Given that a significant number of patients with CRSwNP achieve disease control through appropriate surgical treatments, combined with the use of high-volume topical corticosteroid lavage, the early use of biologics, except for patients with contraindications for surgical procedures, should not be considered the first option for these patients. As per consensus in the therapeutic plan, patients should have undergone at least one appropriate sinonasal surgery, emphasizing the importance of a clear understanding of this definition.

The appropriate surgery, regardless of the technique adopted, should result in the wide opening of the maxillary, ethmoid, and sphenoid sinuses. The frontal sinus should be accessible through at least one Draf IIa-type approach, but less favorable anatomies may necessitate more extended accesses.

The adequate control of inflammatory disease in sinuses where medication lavage easily reaches the mucosa (such as the maxillary and sphenoid sinuses), but not in more challenging locations (such as the frontal sinus), may suggest that a broader surgery should be considered as a complementary treatment option. On the other hand, early recurrences of polyps (<6-months), despite appropriate topical treatment, indicate that a new surgery would have a lower likelihood of success.

In other words, there is still a long way to go, from the necessary explanation to patients with CRSwNP about the existence of biologics until their actual prescription. This is attributed to both the lack of information on cost-effectiveness and the relatively short follow-up time for confirming long-term safety. Regardless of the therapeutic option (or options) chosen, patients with type 2 CRSwNP need to be educated about the importance of their active participation for the success of the treatment.

## Pragmatic proposal and cost-effectiveness

Our previous guideline originally established a minimum scoring system based on clinical (endoscopic, quality of life, and olfaction scores), serological, anatomopathological, and radiological aspects to determine whether patients with CRSwNP are eligible or not for biologic prescription. There needs to be a collective awareness within the medical community regarding the importance of striving to provide patients with the best therapeutic options, taking into consideration the potential risks, benefits, and costs to society. Financial aspects and the lack of adequate response in at least 30% of patients^2^ require adherence to specific criteria when determining the prescription of such medications.

The field of medicine is dynamic, and the time span between updates of guidelines like this should become increasingly shorter. It is possible that improvements in the cost-effectiveness of biologics may alter the prioritization of therapeutic options.

## Conflicts of interest

The authors declare no conflicts of interest.

## References

[1] DeConde A.S., Mace J.C., Levy J.M., Rudmik L., Alt J.A., Smith T.L. (2017). Prevalence of polyp recurrence after endoscopic sinus surgery for chronic rhinosinusitis with nasal polyposis. Laryngoscope..

[2] Fokkens W.J., Lund V.J., Hopkins C., Hellings P.W., Kern R., Reitsma S. (2020). European position paper on rhinosinusitis and nasal polyps 2020. Rhinology..

[3] Damask C.C., Ryan M.W., Casale T.B., Castro M., Franzese C.B., Lee S.E. (2021). Targeted molecular therapies in allergy and rhinology. Otolaryngol Head Neck Surg..

[4] Lloyd C.M., Snelgrove R.J. (2018). Type 2 immunity: expanding our view. Sci Immunol..

[5] Grayson J.W., Hopkins C., Mori E., Senior B., Harvey R.J. (2020). Contemporary classification of chronic rhinosinusitis beyond polyps vs no polyps: a review. JAMA Otolaryngol Head Neck Surg..

[6] Gandhi N.A., Bennett B.L., Graham N.M.H., Pirozzi G., Stahl N., Yancopoulos G.D. (2016). Targeting key proximal drivers of type 2 inflammation in disease. Nat Rev Drug Discov..

[7] Laidlaw T.M., Buchheit K.M. (2020). Biologics in chronic rhinosinusitis with nasal polyposis. Ann Allergy Asthma Immunol..

[8] Marcus S., Schertzer J., Roland L.T., Wise S.K., Levy J.M., DelGaudio J.M. (2020). Central compartment atopic disease: prevalence of allergy and asthma compared with other subtypes of chronic rhinosinusitis with nasal polyps. Int Forum Allergy Rhinol..

[9] Kowalski M.L., Agache I., Bavbek S., Bakirtas A., Blanca M., Bochenek G. (2019). Diagnosis and management of NSAID-Exacerbated Respiratory Disease (N-ERD)-a EAACI position paper. Allergy..

[10] Taniguchi M., Mitsui C., Hayashi H., Ono E., Kajiwara K., Mita H. (2019). Aspirin-exacerbated respiratory disease (AERD): current understanding of AERD. Allergol Int..

[11] Bent J.P., Kuhn F.A. (1994). Diagnosis of allergic fungal sinusitis. Otolaryngol Head Neck Surg..

[12] Grayson P.C., Ponte C., Suppiah R., Robson J.C., Craven A., Judge A. (2022). 2022 American College of Rheumatology/European Alliance of Associations for Rheumatology Classification Criteria for Eosinophilic Granulomatosis With Polyangiitis. Arthritis Rheumatol..

[13] Fokkens W.J., Lund V., Bachert C., Mullol J., Bjermer L., Bousquet J. (2019). EUFOREA consensus on biologics for CRSwNP with or without asthma. Allergy.

[14] Leavitt R.Y., Fauci A.S., Bloch D.A., Michel B.A., Hunder G.G., Arend W.P. (1990). The American College of Rheumatology 1990 criteria for the classification of Wegener’s granulomatosis. Arthritis Rheum..

[15] Casale T.B. (2017). Biologics and biomarkers for asthma, urticaria, and nasal polyposis. J Allergy Clin Immunol..

[16] Codispoti C.D., Mahdavinia M. (2019). A call for cost-effectiveness analysis for biologic therapies in chronic rhinosinusitis with nasal polyps. Ann Allergy Asthma Immunol..

[17] Bang L.M., Plosker G.L. (2004). Omalizumab: a review of its use in the management of allergic asthma. Treat Respir Med..

[18] Kartush A.G., Schumacher J.K., Shah R., Patadia M.O. (2019). Biologic agents for the treatment of chronic rhinosinusitis with nasal polyps. Am J Rhinol Allergy..

[19] Xolair® (omalizumabe). Portal Novartis. 2021. https://portal.novartis.com.br/medicamentos/xolair/. [Accessed 08 December 2023].

[20] Kim H., Ellis A.K., Fischer D., Noseworthy M., Olivenstein R., Chapman K.R. (2017). Asthma biomarkers in the age of biologics. Allergy Asthma Clin Immunol..

[21] Mostafa B.E., Fadel M., Mohammed M.A., Hamdi T.A.H., Askoura A.M. (2020). Omalizumab versus intranasal steroids in the post-operative management of patients with allergic fungal rhinosinusitis. Eur Arch Otorhinolaryngol..

[22] Lowe P.J., Tannenbaum S., Gautier A., Jimenez P. (2009). Relationship between omalizumab pharmacokinetics, IgE pharmacodynamics and symptoms in patients with severe persistent allergic (IgE-mediated) asthma. Br J Clin Pharmacol..

[23] Hayashi H., Mitsui C., Nakatani E., Fukutomi Y., Kajiwara K., Watai K. (2016). Omalizumab reduces cysteinyl leukotriene and 9α,11β-prostaglandin F2 overproduction in aspirin-exacerbated respiratory disease. J Allergy Clin Immunol..

[24] Aksu K., Kurt E. (2013). Aspirin tolerance following omalizumab therapy in a patient with aspirin-exacerbated respiratory disease. Allergol Immunopathol..

[25] Bobolea I., Barranco P., Fiandor A., Cabañas R., Quirce S. (2010). Omalizumab: a potential new therapeutic approach for aspirin-exacerbated respiratory disease. J Investig Allergol Clin Immunol..

[26] Bachert C., Zhang L., Gevaert P. (2015). Current and future treatment options for adult chronic rhinosinusitis: Focus on nasal polyposis. J Allergy Clin Immunol..

[27] Gevaert P., Calus L., Van Zele T., Blomme K., De Ruyck N., Bauters W. (2013). Omalizumab is effective in allergic and nonallergic patients with nasal polyps and asthma. J Allergy Clin Immunol..

[28] Pinto J.M., Mehta N., DiTineo M., Wang J., Baroody F.M., Naclerio R.M. (2010). A randomized, double-blind, placebo-controlled trial of anti-IgE for chronic rhinosinusitis. Rhinology..

[29] Rivero A., Liang J. (2017). Anti-IgE and Anti-IL5 biologic therapy in the treatment of nasal polyposis: a systematic review and meta-analysis. Ann Otol Rhinol Laryngol..

[30] Tsetsos N., Goudakos J.K., Daskalakis D., Konstantinidis I., Markou K. (2018). Monoclonal antibodies for the treatment of chronic rhinosinusitis with nasal polyposis: a systematic review. Rhinology..

[31] Hong C.J., Tsang A.C., Quinn J.G., Bonaparte J.P., Stevens A., Kilty S.J. (2015). Anti-IgE monoclonal antibody therapy for the treatment of chronic rhinosinusitis: a systematic review. Syst Rev..

[32] Santos T.S., Certal V.F., Gonçalves P., Carvalho C. (2014). Effectiveness of omalizumab in the treatment of chronic rhinosinusitis with nasal polyps: systematic review. Eur Sci J..

[33] Gevaert P., Omachi T.A., Corren J., Mullol J., Han J., Lee S.E. (2020). Efficacy and safety of omalizumab in nasal polyposis: 2 randomized phase 3 trials. J Allergy Clin Immunol..

[34] Gevaert P., Saenz R., Corren J., Han J.K., Mullol J., Lee S.E. (2022). Long-term efficacy and safety of omalizumab for nasal polyposis in an open-label extension study. J Allergy Clin Immunol..

[35] Damask C., Chen M., Holweg C.T.J., Yoo B., Millette L.A., Franzese C. (2022). Defining the efficacy of omalizumab in nasal polyposis: A POLYP 1 and POLYP 2 subgroup analysis. Am J Rhinol Allergy..

[36] Maza-Solano J., Callejon-Leblic A., Martin-Jimenez D., Moreno-Luna R., Gonzalez-Garcia J., Cuvillo A. (2023). Omalizumab treatment in uncontrolled asthma and CRSwNP patients, with previous endoscopic sinus surgery, to improve quality of life and endoscopic outcomes: a Two-Year Real-Life Study. Curr Allergy Asthma Rep..

[37] Bachert C., Gevaert P., Hellings P. (2017). Biotherapeutics in chronic rhinosinusitis with and without nasal polyps. J Allergy Clin Immunol Pract..

[38] Humbert M., Busse W., Hanania N.A., Lowe P.J., Canvin J., Erpenbeck V.J. (2014). Omalizumab in asthma: an update on recent developments. J Allergy Clin Immunol Pract..

[39] Jachiet M., Samson M., Cottin V., Kahn J.-E., Le Guenno G., Bonniaud P. (2016). Anti-IgE monoclonal antibody (Omalizumab) in refractory and relapsing eosinophilic granulomatosis with polyangiitis (Churg-Strauss): data on seventeen patients. Arthritis Rheumatol..

[40] Gan E.C., Habib A.-R.R., Rajwani A., Javer A.R. (2015). Omalizumab therapy for refractory allergic fungal rhinosinusitis patients with moderate or severe asthma. Am J Otolaryngol..

[41] Long A., Rahmaoui A., Rothman K.J., Guinan E., Eisner M., Bradley M.S. (2014). Incidence of malignancy in patients with moderate-to-severe asthma treated with or without omalizumab. J Allergy Clin Immunol..

[42] Sanderson C.J. (1992). Interleukin-5, eosinophils, and disease. Blood..

[43] Gevaert P., Bachert C., Holtappels G., Novo C.P., Van der Heyden J., Fransen L. (2003). Enhanced soluble interleukin-5 receptor alpha expression in nasal polyposis. Allergy..

[44] Gevaert P., Van Bruaene N., Cattaert T., Van Steen K., Van Zele T., Acke F. (2011). Mepolizumab, a humanized anti-IL-5 mAb, as a treatment option for severe nasal polyposis. J Allergy Clin Immunol..

[45] Simon H.U., Yousefi S., Schranz C., Schapowal A., Bachert C., Blaser K. (1997). Direct demonstration of delayed eosinophil apoptosis as a mechanism causing tissue eosinophilia. J Immunol..

[46] Nucala (mepolizumab) Label FDA. Fda.gov. 2017. https://www.accessdata.fda.gov/drugsatfda_docs/label/2015/125526Orig1s000Lbl.pdf. [Accessed December 08 2023].

[47] Cinqair (reslizumab) Label FDA. Fda.gov. 2017. https://www.accessdata.fda.gov/drugsatfda_docs/label/2016/761033lbl.pdf. [Accessed December 08 2023].

[48] Fasenra (benralizumab). Fda.gov. 2017. https://www.astrazeneca.com/media-centre/press-releases/2017/fasenra-benralizumab-receives-us-fda-approval-for-severe-uncontrolled-eosinophilic-asthma-14112017.html. [Accessed December 08 2023].

[49] Leckie M.J., ten Brinke A., Khan J., Diamant Z., O’Connor B.J., Walls C.M. (2000). Effects of an interleukin-5 blocking monoclonal antibody on eosinophils, airway hyper-responsiveness, and the late asthmatic response. Lancet..

[50] Castro M., Mathur S., Hargreave F., Boulet L.-P., Xie F., Young J. (2011). Reslizumab for poorly controlled, eosinophilic asthma: a randomized, placebo-controlled study. Am J Respir Crit Care Med..

[51] Pavord I.D., Korn S., Howarth P., Bleecker E.R., Buhl R., Keene O.N. (2012). Mepolizumab for severe eosinophilic asthma (DREAM): a multicentre, double-blind, placebo-controlled trial. Lancet..

[52] Farne H.A., Wilson A., Powell C., Bax L., Milan S.J. (2017). Anti-IL5 therapies for asthma. Cochrane Database Syst Rev..

[53] Harvey E.S., Langton D., Katelaris C., Stevens S., Farah C.S., Gillman A. (2020). Mepolizumab effectiveness and identification of super-responders in severe asthma. Eur Respir J..

[54] Pavord I.D., Bel E.H., Bourdin A., Chan R., Han J.K., Keene O.N. (2022). From DREAM to REALITI-A and beyond: mepolizumab for the treatment of eosinophil-driven diseases. Allergy..

[55] Charles D., Shanley J., Temple S.-N., Rattu A., Khaleva E., Roberts G. (2022). Real-world efficacy of treatment with benralizumab, dupilumab, mepolizumab and reslizumab for severe asthma: a systematic review and meta-analysis. Clin Exp Allergy..

[56] Gevaert P., Lang-Loidolt D., Lackner A., Stammberger H., Staudinger H., Van Zele T. (2006). Nasal IL-5 levels determine the response to anti-IL-5 treatment in patients with nasal polyps. J Allergy Clin Immunol..

[57] Boiko N.V., Lodochkina O.E., Kit M.M., Kuleshova V.G., Nedashkovskaya N.G. (2021). Impact of reslizumab on the course of chronic rhinosinusitis in patients with eosinophilic asthma. Vestn Otorinolaringol..

[58] Bachert C., Han J.K., Desrosiers M.Y., Gevaert P., Heffler E., Hopkins C. (2022). Efficacy and safety of benralizumab in chronic rhinosinusitis with nasal polyps: a randomized, placebo-controlled trial. J Allergy Clin Immunol..

[59] Han J.K., Bachert C., Fokkens W., Desrosiers M., Wagenmann M., Lee S.E. (2021). Mepolizumab for chronic rhinosinusitis with nasal polyps (SYNAPSE): a randomised, double-blind, placebo-controlled, phase 3 trial. Lancet Respir Med..

[60] Bachert C., Sousa A.R., Lund V.J., Scadding G.K., Gevaert P., Nasser S. (2017). Reduced need for surgery in severe nasal polyposis with mepolizumab: randomized trial. J Allergy Clin Immunol..

[61] Kim C., Han J., Wu T., Bachert C., Fokkens W., Hellings P. (2021). Role of biologics in chronic rhinosinusitis with nasal polyposis: state of the art review. Otolaryngol Head Neck Surg..

[62] Tversky J., Lane A.P., Azar A. (2021). Benralizumab effect on severe chronic rhinosinusitis with nasal polyps (CRSwNP): a randomized double-blind placebo-controlled trial. Clin Exp Allergy..

[63] Chupp G., Alobid I., Lugogo N.L., Kariyawasam H.H., Bourdin A., Chaker A.M. (2023). Mepolizumab reduces systemic corticosteroid use in chronic rhinosinusitis with nasal polyps. J Allergy Clin Immunol Pract..

[64] Silver J., Deb A., Laliberté F., Gao C., Bhattacharyya N. (2023). Real-world effectiveness of mepolizumab in severe asthma and chronic rhinosinusitis in the United States: impact of comorbidity and sinus surgery. Int Forum Allergy Rhinol..

[65] Domínguez-Sosa M.S., Cabrera-Ramírez M.S., Marrero-Ramos M.D.C., Dávila-Quintana D., Cabrera-López C., Carrillo-Díaz T. (2023). Real-life effectiveness of mepolizumab in refractory chronic rhinosinusitis with nasal polyps. Biomedicines..

[66] United States Food and Drug Administration. Dupixent Prescribing Information. 2017. https://www.accessdata.fda.gov/drugsatfda_docs/label/2019/761055s014lbl.pdf [Accessed November 30 2020].

[67] European Medicines Agency. Dupixent Summary of Product Characteristics. https://www.ema.europa.eu/en/medicines/human/EPAR/dupixent. [Accessed November 30 2020].

[68] Agência Nacional de Vigilância Sanitária Consultas Anvisa Medicamentos Dupixent. https://consultas.anvisa.gov.br/#/medicamentos/25351189487201920/?nomeProduto=dupixent. [Accessed November 30 2020].

[69] Dunican E.M., Fahy J.V. (2015). The role of type 2 inflammation in the pathogenesis of asthma exacerbations. Ann Am Thorac Soc..

[70] Sastre J., Dávila I. (2018). Dupilumab: a new paradigm for the treatment of allergic diseases. J Investig Allergol Clin Immunol..

[71] Bachert C., Laidlaw T.M., Cho S.H., Mullol J., Swanson B.N., Naimi S. (2023). Effect of Dupilumab on type 2 biomarkers in chronic rhinosinusitis with nasal polyps: SINUS-52 study results. Ann Otol Rhinol Laryngol..

[72] Wenzel S., Ford L., Pearlman D., Spector S., Sher L., Skobieranda F. (2013). Dupilumab in persistent asthma with elevated eosinophil levels. N Engl J Med..

[73] Wenzel S., Castro M., Corren J., Maspero J., Wang L., Zhang B. (2016). Dupilumab efficacy and safety in adults with uncontrolled persistent asthma despite use of medium-to-high-dose inhaled corticosteroids plus a long-acting β2 agonist: a randomised double-blind placebo-controlled pivotal phase 2b dose-ranging trial. Lancet..

[74] Corren J., Castro M., Chanez P., Fabbri L., Joish V.N., Amin N. (2019). Dupilumab improves symptoms, quality of life, and productivity in uncontrolled persistent asthma. Ann Allergy Asthma Immunol..

[75] Castro M., Corren J., Pavord I.D., Maspero J., Wenzel S., Rabe K.F. (2018). Dupilumab efficacy and safety in moderate-to-severe uncontrolled asthma. N Engl J Med..

[76] Rabe K.F., Nair P., Brusselle G., Maspero J.F., Castro M., Sher L. (2018). Efficacy and safety of Dupilumab in glucocorticoid-dependent severe asthma. N Engl J Med..

[77] Busse W.W., Maspero J.F., Rabe K.F., Papi A., Wenzel S.E., Ford L.B. (2018). Liberty Asthma QUEST: phase 3 randomized, double-blind, placebo-controlled, parallel-group study to evaluate Dupilumab efficacy/safety in patients with uncontrolled, moderate-to-severe asthma. Adv Ther..

[78] Bacharier L.B., Maspero J.F., Katelaris C.H., Fiocchi A.G., Gagnon R., de Mir I. (2021). Dupilumab in children with uncontrolled moderate-to-severe asthma. N Engl J Med..

[79] Wechsler M.E., Ford L.B., Maspero J.F., Pavord I.D., Papi A., Bourdin A. (2022). Long-term safety and efficacy of dupilumab in patients with moderate-to-severe asthma (TRAVERSE): an open-label extension study. Lancet Respir Med..

[80] Bacharier L.B., Maspero J.F., Katelaris C.H., Fiocchi A.G., Gagnon R., de Mir I. (2023). Assessment of long-term safety and efficacy of dupilumab in children with asthma (LIBERTY ASTHMA EXCURSION): an open-label extension study. Lancet Respir Med..

[81] Bachert C. (2016). Innovative therapeutic targets in chronic sinusitis with nasal polyps. Braz J Otorhinolaryngol..

[82] Bachert C., Mannent L., Naclerio R.M., Mullol J., Ferguson B.J., Gevaert P. (2016). Effect of subcutaneous Dupilumab on nasal polyp burden in patients with chronic sinusitis and nasal polyposis: a randomized clinical trial. JAMA..

[83] Bachert C., Han J.K., Desrosiers M., Hellings P.W., Amin N., Lee S.E. (2019). Efficacy and safety of dupilumab in patients with severe chronic rhinosinusitis with nasal polyps (LIBERTY NP SINUS-24 and LIBERTY NP SINUS-52): results from two multicentre, randomised, double-blind, placebo-controlled, parallel-group phase 3 trials. Lancet..

[84] Bachert C., Zinreich S.J., Hellings P.W., Mullol J., Hamilos D.L., Gevaert P. (2020). Dupilumab reduces opacification across all sinuses and related symptoms in patients with CRSwNP. Rhinology..

[85] Jonstam K., Swanson B.N., Mannent L.P., Cardell L.-O., Tian N., Wang Y. (2019). Dupilumab reduces local type 2 pro-inflammatory biomarkers in chronic rhinosinusitis with nasal polyposis. Allergy..

[86] Bachert C., Cho S., Laidlaw T., Swanson B., Harel S., Mannent L. (2020). Dupilumab reduces blood, urine, and nasal biomarkers of type 2 inflammation in patients with chronic rhinosinusitis with nasal polyps in the Phase 3 SINUS-52 trial. J Allergy Clin Immunol..

[87] Bachert C., Hellings P.W., Mullol J., Naclerio R.M., Chao J., Amin N. (2019). Dupilumab improves patient-reported outcomes in patients with chronic rhinosinusitis with nasal polyps and comorbid asthma. J Allergy Clin Immunol Pract..

[88] Weinstein S.F., Katial R., Jayawardena S., Pirozzi G., Staudinger H., Eckert L. (2018). Efficacy and safety of dupilumab in perennial allergic rhinitis and comorbid asthma. J Allergy Clin Immunol..

[89] Peters A.T., Wagenmann M., Bernstein J.A., Khan A.H., Nash S., Jacob-Nara J.A. (2023). Dupilumab efficacy in patients with chronic rhinosinusitis with nasal polyps with and without allergic rhinitis. Allergy Asthma Proc..

[90] Fujieda S., Matsune S., Takeno S., Ohta N., Asako M., Bachert C. (2022). Dupilumab efficacy in chronic rhinosinusitis with nasal polyps from SINUS-52 is unaffected by eosinophilic status. Allergy..

[91] Laidlaw T.M., Mullol J., Fan C., Zhang D., Amin N., Khan A. (2019). Dupilumab improves nasal polyp burden and asthma control in patients with CRSwNP and AERD. J Allergy Clin Immunol Pract..

[92] Bachert C., Hellings P.W., Mullol J., Hamilos D.L., Gevaert P., Naclerio R.M. (2020). Dupilumab improves health-related quality of life in patients with chronic rhinosinusitis with nasal polyposis. Allergy..

[93] Chong L.-Y., Piromchai P., Sharp S., Snidvongs K., Philpott C., Hopkins C. (2020). Biologics for chronic rhinosinusitis. Cochrane Database Syst Rev..

[94] Lo R.M., Liu A.Y., Valdez T.A., Gernez Y. (2020). Dupilumab use in recalcitrant allergic fungal rhinosinusitis. Ann Allergy Asthma Immunol..

[95] van der Lans R.J.L., Fokkens W.J., Adriaensen G.F.J.P.M., Hoven D.R., Drubbel J.J., Reitsma S. (2022). Real-life observational cohort verifies high efficacy of dupilumab for chronic rhinosinusitis with nasal polyps. Allergy..

[96] Mustafa S.S., Vadamalai K., Scott B., Ramsey A. (2021). Dupilumab as add-on therapy for chronic rhinosinusitis with nasal polyposis in aspirin exacerbated respiratory disease. Am J Rhinol Allergy..

[97] De Corso E., Pasquini E., Trimarchi M., La Mantia I., Pagella F., Ottaviano G. (2023). Dupilumab in the treatment of severe uncontrolled chronic rhinosinusitis with nasal polyps (CRSwNP): a multicentric observational Phase IV real-life study (DUPIREAL). Allergy..

[98] Miglani A., Soler Z.M., Smith T.L., Mace J.C., Schlosser R.J. (2023). A comparative analysis of endoscopic sinus surgery versus biologics for treatment of chronic rhinosinusitis with nasal polyposis. Int Forum Allergy Rhinol..

[99] Hopkins C., Wagenmann M., Bachert C., Desrosiers M., Han J.K., Hellings P.W. (2021). Efficacy of dupilumab in patients with a history of prior sinus surgery for chronic rhinosinusitis with nasal polyps. Int Forum Allergy Rhinol..

[100] Scangas G.A., Wu A.W., Ting J.Y., Metson R., Walgama E., Shrime M.G. (2021). Cost utility analysis of Dupilumab versus endoscopic sinus surgery for chronic rhinosinusitis with nasal polyps. Laryngoscope..

[101] Cai S., Xu S., Lou H., Zhang L. (2022). Comparison of different biologics for treating chronic rhinosinusitis with nasal polyps: a network analysis. J Allergy Clin Immunol Pract..

[102] De Prado Gomez L., Khan A.H., Peters A.T., Bachert C., Wagenmann M., Heffler E. (2022). Efficacy and safety of Dupilumab versus Omalizumab in chronic rhinosinusitis with nasal polyps and asthma: EVEREST trial design. Am J Rhinol Allergy..

[103] Mullol J., Laidlaw T.M., Bachert C., Mannent L.P., Canonica G.W., Han J.K. (2022). Efficacy and safety of dupilumab in patients with uncontrolled severe chronic rhinosinusitis with nasal polyps and a clinical diagnosis of NSAID-ERD: results from two randomized placebo-controlled phase 3 trials. Allergy..

[104] Xu X., Reitsma S., Wang D.Y., Fokkens W.J. (2022). Updates in biologic therapy for chronic rhinosinusitis with nasal polyps and NSAID-exacerbated respiratory disease. Allergy..

[105] Wangberg H., Spierling Bagsic S.R., Osuna L., White A.A. (2022). Appraisal of the real-world effectiveness of biologic therapies in aspirin-exacerbated respiratory disease. J Allergy Clin Immunol Pract..

[106] Oykhman P., Paramo F.A., Bousquet J., Kennedy D.W., Brignardello-Petersen R., Chu D.K. (2022). Comparative efficacy and safety of monoclonal antibodies and aspirin desensitization for chronic rhinosinusitis with nasal polyposis: a systematic review and network meta-analysis. J Allergy Clin Immunol..

[107] Sánchez J., García E., Lopez J.-F., Calle A., Buendia J.-A. (2023). Nonsteroidal Anti-inflammatory Drug (NSAID) tolerance after biological therapy in patients with NSAID-Exacerbated Respiratory Disease: a randomized comparative trial. J Allergy Clin Immunol Pract..

[108] Siddiqui S., Bachert C., Chaker A.M., Han J.K., Hellings P.W., Peters A.T. (2022). AROMA: real-world global registry of dupilumab for chronic rhinosinusitis with nasal polyps. ERJ Open Res..

[109] Garvey E., Naimi B., Duffy A., Hannikainen P., Kahn C., Farquhar D. (2023). Optimizing the timing of biologic and surgical therapy for patients with refractory chronic rhinosinusitis with nasal polyposis (CRSwNP). Int Forum Allergy Rhinol..

[110] van der Lans R.J.L., Otten J.J., Adriaensen G.F.J.P.M., Hoven D.R., Benoist L.B., Fokkens W.J. (2023). Two-year results of tapered dupilumab for CRSwNP demonstrates enduring efficacy established in the first 6 months. Allergy..

[111] Akinlade B., Guttman-Yassky E., de Bruin-Weller M., Simpson E.L., Blauvelt A., Cork M.J. (2019). Conjunctivitis in dupilumab clinical trials. Br J Dermatol..

[112] Stack T.J., Kim S., Lamb M.M., Mohammad I., Zeatoun A., Lopez E. (2023). Characterizing adverse events of biologic treatment of T2 disease: a disproportionality analysis of the FDA adverse event reporting system. ORL J Otorhinolaryngol Relat Spec..

[113] Brightling C.E., Chanez P., Leigh R., O’Byrne P.M., Korn S., She D. (2015). Efficacy and safety of tralokinumab in patients with severe uncontrolled asthma: a randomised, double-blind, placebo-controlled, phase 2b trial. Lancet Respir Med..

[114] Sakamoto K., Matsuki S., Irie S., Uchida N., Hayashi N., Horiuchi M. (2020). A Phase 1, randomized, placebo-controlled study to evaluate the safety, tolerability, pharmacokinetics, and immunogenicity of subcutaneous tezepelumab in healthy japanese men. Clin Pharmacol Drug Dev..

[115] Corren J., Karpefors M., Hellqvist Å., Parnes J.R., Colice G. (2021). Tezepelumab Reduces exacerbations across all seasons in patients with severe, uncontrolled asthma: a post hoc analysis of the PATHWAY Phase 2b study. J Asthma Allergy..

[116] Corren J., Parnes J.R., Wang L., Mo M., Roseti S.L., Griffiths J.M. (2017). Tezepelumab in adults with uncontrolled asthma. N Engl J Med..

[117] Menzies-Gow A., Corren J., Bourdin A., Chupp G., Israel E., Wechsler M.E. (2021). Tezepelumab in adults and adolescents with severe, uncontrolled asthma. N Engl J Med..

[118] Pavord I.D., Hoyte F.C.L., Lindsley A.W., Ambrose C.S., Spahn J.D., Roseti S.L. (2023). Tezepelumab reduces exacerbations across all seasons in patients with severe, uncontrolled asthma (NAVIGATOR). Ann Allergy Asthma Immunol..

[119] Zoumot Z., Al Busaidi N., Tashkandi W., Aljohaney A.A., Isse S., Vidyasagar K. (2022). Tezepelumab for patients with severe uncontrolled asthma: a systematic review and meta-analysis. J Asthma Allergy..

[120] Shaban Abdelgalil M., Ahmed Elrashedy A., Awad A.K., Reda Gad E., Ali M.M., Abdelmoez Farahat R. (2022). Safety and efficacy of tezepelumab vs. placebo in adult patients with severe uncontrolled asthma: a systematic review and meta-analysis. Sci Rep..

[121] Corren J., Menzies-Gow A., Chupp G., Israel E., Korn S., Cook B. (2023). Efficacy of tezepelumab in severe, uncontrolled asthma: pooled analysis of the PATHWAY and NAVIGATOR clinical trials. Am J Respir Crit Care Med..

[122] Corren J., Menzies-Gow A., Bimmel J., McGuinness A., Almqvist G., Bowen K. (2023). Tezepelumab for the treatment of severe asthma: a plain language summary of the PATHWAY and NAVIGATOR studies. Immunotherapy..

[123] Chagas G.C.L., Xavier D., Gomes L., Ferri-Guerra J., Oquet R.E.H. (2023). Effects of Tezepelumab on quality of life of patients with moderate-to-severe, uncontrolled asthma: systematic review and meta-analysis. Curr Allergy Asthma Rep..

[124] Emson C., Corren J., Sałapa K., Hellqvist Å., Parnes J.R., Colice G. (2021). Efficacy of tezepelumab in patients with severe, uncontrolled asthma with and without nasal polyposis: a post hoc analysis of the Phase 2b PATHWAY study. J Asthma Allergy..

[125] Laidlaw T.M., Menzies-Gow A., Caveney S., Han J.K., Martin N., Israel E. (2023). Tezepelumab efficacy in patients with severe, uncontrolled asthma with comorbid nasal polyps in NAVIGATOR. J Asthma Allergy..

[126] Delemarre T., Holtappels G., De Ruyck N., Zhang N., Nauwynck H., Bachert C. (2020). Type 2 inflammation in chronic rhinosinusitis without nasal polyps: another relevant endotype. J Allergy Clin Immunol..

[127] Tomassen P., Vandeplas G., Van Zele T., Cardell L.-O., Arebro J., Olze H. (2016). Inflammatory endotypes of chronic rhinosinusitis based on cluster analysis of biomarkers. J Allergy Clin Immunol..

[128] Chiang S., Lee S.E. (2023). New concepts in Barrier dysfunction in CRSwNP and emerging roles of tezepelumab and dupilumab. Am J Rhinol Allergy..

[129] Striz I., Golebski K., Strizova Z., Loukides S., Bakakos P., Hanania N.A. (2023). New insights into the pathophysiology and therapeutic targets of asthma and comorbid chronic rhinosinusitis with or without nasal polyposis. Clin Sci..

[130] Chung S.A., Langford C.A., Maz M., Abril A., Gorelik M., Guyatt G. (2021). 2021 American College of Rheumatology/Vasculitis Foundation Guideline for the Management of Antineutrophil Cytoplasmic Antibody-Associated Vasculitis. Arthritis Rheumatol..

[131] Caminati M., Maule M., Bello F., Emmi G. (2023). Biologics for eosinophilic granulomatosis with polyangiitis. Curr Opin Allergy Clin Immunol..

[132] Portacci A., Campisi R., Buonamico E., Nolasco S., Pelaia C., Crimi N. (2023). Real-world characteristics of “super-responders” to mepolizumab and benralizumab in severe eosinophilic asthma and eosinophilic granulomatosis with polyangiitis. ERJ Open Res..

[133] Unizony S., Villarreal M., Miloslavsky E.M., Lu N., Merkel P.A., Spiera R. (2016). Clinical outcomes of treatment of anti-neutrophil cytoplasmic antibody (ANCA)-associated vasculitis based on ANCA type. Ann Rheum Dis..

[134] Charles P., Perrodeau É., Samson M., Bonnotte B., Néel A., Agard C. (2020). Long-Term Rituximab use to maintain remission of antineutrophil cytoplasmic antibody-associated vasculitis: a randomized trial. Ann Intern Med..

[135] Puéchal X., Iudici M., Calich A.L., Vivot A., Terrier B., Régent A. (2019). Rituximab for induction and maintenance therapy of granulomatosis with polyangiitis: a single-centre cohort study on 114 patients. Rheumatology..

[136] Ho C., Spry C. (2017).

[137] Ahn S.S., Lee S.-W. (2023). Management of antineutrophil cytoplasmic antibody-associated vasculitis: a review of recent guidelines. J Rheum Dis..

[138] Schirmer J.H., Sanchez-Alamo B., Hellmich B., Jayne D., Monti S., Luqmani R.A. (2023). Systematic literature review informing the 2022 update of the EULAR recommendations for the management of ANCA-associated vasculitis (AAV): part 1-treatment of granulomatosis with polyangiitis and microscopic polyangiitis. RMD Open..

[139] Yates M., Watts R.A., Bajema I.M., Cid M.C., Crestani B., Hauser T. (2016). EULAR/ERA-EDTA recommendations for the management of ANCA-associated vasculitis. Ann Rheum Dis..

[140] Anselmo-Lima W.T., Tamashiro E., Romano F.R., Miyake M.M., Roithmann R., Kosugi E.M. (2022). Guideline for the use of immunobiologicals in chronic rhinosinusitis with nasal polyps (CRSwNP) in Brazil. Braz J Otorhinolaryngol..

[141] Landis B.N., Hummel T., Hugentobler M., Giger R., Lacroix J.S. (2003). Ratings of overall olfactory function. Chem Senses..

[142] Landis B.N., Hummel T. (2020). Measuring olfaction instead of asking: it is more than luxury! Eur. Arch. Otorhinolaryngol..

[143] Oleszkiewicz A., Hummel T. (2019). Whose nose does not know? Demographical characterization of people unaware of anosmia. Eur Arch Otorhinolaryngol..

[144] Fornazieri M.A., dos Santos C.A., Bezerra T.F.P., Pinna F de R., Voegels R.L., Doty R.L. (2015). Development of normative data for the Brazilian adaptation of the University of Pennsylvania Smell Identification Test. Chem Senses..

[145] Fenólio G.H.M., Anselmo-Lima W.T., Tomazini G.C., Compagnoni I.M., Amaral M.S.A. do, Fantucci M.Z. (2022). Validation of the Connecticut olfactory test (CCCRC) adapted to Brazil. Braz J Otorhinolaryngol..

[146] Haxel B.R., Hummel T., Fruth K., Lorenz K., Gunder N., Nahrath P. (2022). Real-world-effectiveness of biological treatment for severe chronic rhinosinusitis with nasal polyps. Rhinology..

[147] London B., Nabet B., Fisher A.R., White B., Sammel M.D., Doty R.L. (2008). Predictors of prognosis in patients with olfactory disturbance. Ann Neurol..

[148] Doty R.L., Yousem D.M., Pham L.T., Kreshak A.A., Geckle R., Lee W.W. (1997). Olfactory dysfunction in patients with head trauma. Arch Neurol..

[149] Mullol J., Bachert C., Amin N., Desrosiers M., Hellings P.W., Han J.K. (2022). Olfactory outcomes with dupilumab in chronic rhinosinusitis with nasal polyps. J Allergy Clin Immunol Pract..

[150] Cantone E., De Corso E., Ricciardiello F., Di Nola C., Grimaldi G., Allocca V. (2022). Olfaction recovery following dupilumab is independent of nasal polyp reduction in CRSwNP. J Pers Med..

[151] Wu Q., Zhang Y., Kong W., Wang X., Yuan L., Zheng R. (2022). Which is the best biologic for nasal polyps: dupilumab, omalizumab, or mepolizumab? A network meta-analysis. Int Arch Allergy Immunol..

[152] Peters A.T., Han J.K., Hellings P., Heffler E., Gevaert P., Bachert C. (2021). Indirect treatment comparison of biologics in chronic rhinosinusitis with nasal polyps. J Allergy Clin Immunol Pract..

[153] Nakanishi M., Fornazieri M.A., Lança Gomes P., Dias L.A. de M., Freire G.S.M., Vinha L.G. do A. (2022). The digital scent device as a new concept for olfactory assessment. Int Forum Allergy Rhinol..

[154] Toma S., Hopkins C. (2016). Stratification of SNOT-22 scores into mild, moderate or severe and relationship with other subjective instruments. Rhinology..

[155] Chan Y., Thamboo A.V., Han J.K., Desrosiers M. (2023). Remission: does it already exist in chronic rhinosinusitis with nasal polyposis?. J Otolaryngol Head Neck Surg..

[156] Venkatesan P. (2023). 2023 GINA report for asthma. Lancet Respir Med..

